# Women in International Elite Athletics: Gender (in)equality and National Participation

**DOI:** 10.3389/fspor.2021.709640

**Published:** 2021-08-27

**Authors:** Henk Erik Meier, Mara Verena Konjer, Jörg Krieger

**Affiliations:** ^1^Institute of Sport and Exercise Sciences, University of Münster, Münster, Germany; ^2^Department of Public Health, Aarhus University, Aarhus, Denmark

**Keywords:** international athletics, gender inequality, women's empowerment, development programs, multi-level analyses

## Abstract

Gender discrimination has been strongly related to the suppression of women's participation in sport. Accordingly, gender (in)equality has proven to be an important determinant for the participation and the success of countries in international women's elite sport. Hence, differences in gender (in)equalitity, such as women's participation in the labor force, fertility rates, tradition of women suffrage or socio-economic status of women, could be linked to success in international women's elite sports. While major international sport governing bodies have created programs to subsidize the development of women's sports in member countries, gender equality has figured rather low within the International Amateur Athletics Federation (IAAF) (now World Athletics). Therefore, the paper examines the impact of gender (in)equality on country participation in international athletics on the base of a unique dataset on season's bests. The results provide further support that gender inequality matters and is associated with participation in women's elite sports. Whereas, women's participation in athletics has made considerable progress in the past two decades as a side-effect of the IAAF's decentralization strategy, the analyses illustrate the need for better targeted and better resourced development programs for increasing participation of less gender equal countries. Moreover, the analyses indicate the limitations of a pure macro-social approach as there are some rather unexpected dynamic developments, such as, the substantial progress of women's athletics in the Islamic Republic of Iran as a country with strong Muslim religious affiliation. The results from this analysis were used to provide practical implications.

## Introduction

Since men's control of women's physical activity has been at the heart of masculine hegemony, sports has been a highly gendered social sphere. For a long time, women were denied the right to engage in physical exercise for reasons of health, that is, the alleged physical “weakness” of women's bodies or detrimental effects on the fertility of women, chastity or threats to the “natural order” of sexes (e.g., Pfister, 1993; Meier, 2020). Over the last decades, women have made considerable progress with regard to participation in mass sports as well as elite sports. Nevertheless, there is still evidence that sport continues to be gendered. Thus, a persistent finding of macro-social research on international elite sport participation is that the participation and success of women in international elite sports is strongly related to national gender regimes.

International sport governing bodies, such as the International Olympic Committee (IOC) and the international governing body of football (Fédération Internationale de Football Association—FIFA), have tried to promote women's sports and women's sport participation. Such efforts do not necessarily indicate that these organizations have ceased to be institutions of men's hegemony (Fink, 2008; Williams, 2014). Initiatives to promote women's sport might simply reflect the search for new customers in an increasingly saturated sports entertainment market. Nevertheless, there is evidence that such promotional efforts inspired more women's elite sport participation (e.g., Jacobs, 2014).

In contrast, the International Athletics Association Federation (IAAF)—since 2019 known as World Athletics—made little effort to promote women's athletics throughout its history (Krieger, 2021). Therefore, the current paper explores the relationship between gender (in)equality and country participation in women's elite athletics. It does so on the base of a unique dataset on season's best in women's athletics covering the period between 2000 and 2019.

## Theoretical Background

Gender discrimination in international elite sports has been examined from different theoretical and methodological perspectives. Much of the research has more or less characterized women's access to elite sport as the political outcome of a liberal-feminist discourse centering on equal opportunities, socialization practices and legal or institutional reform (e.g., Scraton et al., 1999).

Historical research on women's sport has highlighted how women have been kept out of sport for medical, aesthetic and social rationales (Guttmann, 1991; Hargreaves, 1994; Schultz, 2018). The founder of the modern Olympic Movement, Pierre de Coubertin, thought women's sport was “impractical, uninteresting, ungainly, and, I do not hesitate to add, improper” (Coubertin, 1912). Following attempts to restrict women's participation in the early Olympic Games, more women's events were added during the interwar years due to the growing significance of women's sports and the increasing activities of women's sport organizations (Pfister, 1993). Put simply, men wanted to maintain control over women's sport so it would not exceed the men's sport in popularity (Krieger and Krech, 2020).

After the Second World War, social, economic and legislative changes catalyzed the increased participation of women in elite sport. Between the 1970's and the 1990's, the international women's sport movement gained increasing momentum that culminated in the inaugural World Conference on Women and Sport, held in Brighton in 1994 (Hargreaves, 1999). The outcome of the conference was an international treaty to support the development of a gender equal sport and physical activity system (Brighton Declaration on Women Sport, 1994). The IOC supported and signed what became known as the “Brighton Declaration.” Thus, since the end of the 19th century, women have gained access to participate in all sporting disciplines at the Olympic Games. The 2018 Winter Olympic Games in Pyeongchang were the first Olympics at which more medal events for women than for men were held (IOC, 2020). However, it should be mentioned while women have access to all sporting disciplines in the Olympics, there are still some events which they cannot compete in. In athletics, until 2017 women could only participate in 20 km race walk, but not in the 50 km race walk.

The current study does, however, not focus on the women's sport movement's struggle to gain access to elite sports but examines the (relative) impact of national gender regimes on country participation in international elite sport. The concept of gender regimes tries to grasp gender hierarchy within societies. According to the influential contribution of Connell (2002, p. 53–68), a gender regime can be characterized via four dimensions:

- “Gender division of labor,” that is, the way in which production and consumption are arranged along gender lines;
- “Gender relations of power,” that is, the way in which control, authority, and force are exercised along gender lines;
- “Emotion and human relations,” that is, the way in which attachment and antagonism among people and groups are organized along gender lines; and
- “Gender culture and symbolism,” that is, the way in which gender identities are defined in culture, the language and symbols of gender difference, and the prevailing beliefs and attitudes about gender.

The macro-social research on the impact of national gender regimes on country participation has, however, usually not employed such an encompassing definition of gender regimes but focused on gender equality in the spheres of education, labor market and political process (see below). Most of this research is inspired by the parsimonious economic model developed by Bernard and Busse (2004). Accordingly, the production of athletic success can be explained by two primary factors, that is, population size and national wealth. Population size defines the national pool of athletic talents, while national wealth provides the economic means to develop these very talents. Most empirical accounts also consider (former) membership in the communist bloc as additional variable, which has served as a proxy either for organizational capacities or for policy priorities in favor of elite sports policies (Bernard and Busse, 2004). Macro-social research on women's international elite sports has expanded the basic economic model by adding different proxies for gender inequality. In a groundbreaking paper, Klein (2004) demonstrated that stronger participation of women in the labor force related to better women's performances in the Summer Olympics and the Women's Football World Cup even when the analyses controlled for income per capita and population size. Klein's (2004) contribution inspired a vibrant research, which used different indicators of gender inequality but supported his main findings.

With regard to international women's football, Hoffmann et al. (2006) found that the ratio of average women's earnings to men's earning related significantly to better team performances measured by the scores awarded to national women's soccer teams by FIFA's ranking system. Hence, the lower the gender pay gap, the better national team performances. In an ambitious article, which compared determinants of men's and women's team performances as measured by FIFA scores, Congdon- Hohman and Matheson (2013) used the ratio of women's to men's secondary enrollment rates as an indicator for gender equality. They found that the influence of economic and demographic factors were similar for men's and women's team performances. In contrast, Muslim religious affiliation correlated with lower women's success but not men's, while communist political systems showed better women's performances but men's performances were worse. The gender equality indicator used seemed to exert a positive impact on women's soccer performance but not on men's. Cho (2013) also used FIFA scores to examine the question whether football traditions or women empowerment were a driving force for national success in women's soccer. Again women's labor force participation served as proxy for gender equality. Cho (2013) found that women's empowerment correlated with the success in women's soccer.

Concerning success in the Olympics, Leeds and Leeds (2012) confirmed Klein's (2004) finding that higher women's labor force participation related to improved women's performances at the Summer Olympics. Moreover, they found that lower fertility rates and a longer tradition of women's suffrage also correlated with better women's performances. Noland and Stahler (2016) used several indicators for gender equality in their more recent analyses of women's performances at the Summer Olympics and demonstrated that the socio-economic status of women correlated significantly with better performances. Lowen et al. (2016) employed the gender inequality value (GIV) as developed by the United Nations as predictor for success in the Summer Olympics. They confirmed that greater gender equality has been consistently and significantly associated with improvements in two measures of Olympic success, that is, athletic participation and medal counts, even when other important predictors were taken into account. Interestingly, they even found that higher gender inequality related to lower number of medals won by both men and women. Finally, the finding that Islamic religion is a negative correlate of sporting success in the Olympics has been related to the fact that Islamic religion does not support women's sport participation (Sfeir, 1985; Tcha and Pershin, 2003; Trivedi and Zimmer, 2014; Noland and Stahler, 2016).

These findings can be summarized as follows: There is solid and consistent evidence that macro-social gender inequality relates to women's participation and success in international elite sports. However, the cited macro-social approaches suffer from a number of limitations. With regard to measuring gender (in)equality, the studies exclusively employ macro-social indicators focusing on what has been called “public sphere gender equality,” which refers to women's equality in education, labor market and political process. However, it has been argued that the gender revolution will only be complete when gender equality reaches the private sphere since even in societies with high public sphere gender equality responsibility for household chores is unequally distributed (England, 2010; see also: McDonald, 2000a,b). A second limitation is that most studies fail to consider meso-level factors, “such as sports federations and sports clubs, families, the media, schools and peer groups [which] function as gatekeepers and mediate or moderate the effect of macro-level gender equality” (Lagaert and Roose, 2018, p. 546). Yet, a recent study by Meier (2020) on women's soccer in reunified Germany indicated that macro-social gender equality does not translate in a linear manner into more women's sport participation and that policy priorities of sport organizations at different levels (national, regional and local) appeared to be highly consequential for women's sport participation and the popularity of women's sports. Finally, there is a lack of studies examining the impact of the efforts of international sport governing bodies to promote women's elite sports and to inspire women's sport participation. A particular exception is the innovative study conducted by Jacobs (2014). Jacobs (2014) evaluated the effects of FIFA programs for promoting women's soccer by using FIFA scores as dependent variable. At the macro level, she found income per capita, women's population size and women's labor force participation to be consistently and positively associated with women's team success. In addition, there was a significant impact of meso-level organizational factors on women's team performances. Dedicated governance staff and training proved to be key correlates of successful women's soccer nations in the short term, while dedicated governance staff and investments in youth developments were strong predictors of success in the long term (Jacobs, 2014).

Hence, although the current study follows the path of previous macro-social research on the relationship between gender (in)equality and country participation, it is fully aware of the conceptual and measurement limitations of such an approach. The main innovative contribution of the current study is, therefore, to apply macro-social research approaches to a new subject, that is, country participation in international women's athletics. As will be elaborated now, women have been long marginalized in international athletics.

## Gender Discrimination in International Athletics

The IAAF was founded in 1912 to organize men's international athletics, and initially expressed little interest in the women's sport. It was not until French sport official and feminist Alice Milliat through her organisation Fédération Sportive Féminine Internationale (FSFI) began successfully organizing international athletics competitions for women. In response, the IAAF began to consider extending its influence to cover women athletes. Viewing the FSFI as a threat to its singular authority over the sport, the men's federation usurped control from the women's federation through a series of strategic maneuvers. In 1922, the then President of the IAAF, Sigfrid Edström, ordered the all men's IAAF officials to study the possibility of the IAAF governing women's sport. As result of these efforts, two women's FSFI representatives were co-opted, which contributed to the disintegration of the FSFI. Yet, the influence of the former FSFI representatives was intentionally limited (Krieger and Krech, 2020). A similar development occurred later in the U.S., when the Association for Intercollegiate Athletics for Women (AIAW) was forced to discontinue its activities in 1982 in favor of the NCAA, which until then had been responsible only for men's sports (Wushanley, 2004). When IAAF business resumed after World War II, an all-men's Women's Commission was appointed (IAAF, 1946). It took 10 years before Zoya Romanova was elected as the first women to chair (IAAF, 1956). Moreover, Romanova's recruitment seems to have been primarily motivated by the Soviet demands for greater representation in IAAF leadership positions (Krieger and Duckworth, 2020).

The Women's Commission focused on adding women's events to the athletics programme at the Olympic Games and European Championships. However, progress was rather slow and women's influence in the IAAF's governance structures remained limited. Within the IAAF a centralized power structure and misogynistic culture were deeply intertwined, and characterized the organizational environment within which the Women's Committee operated at least until the early 2000's. For example, in 2002 women still only made up an average of 7.1% of all committee and commission members (outside the Women's Committee) (Bechthold, 2002a,b).

Therefore, the concerns of women's athletes and its development had a difficult stance within the IAAF. Throughout the 1990's, the Women's Committee under the leadership of German sport administrator Ilse Bechthold continued to seek the expansion of the women's programme of events at international competitions. It also adopted the explicit goal that, by the turn of the century, the IAAF should recognize an equal number of events for women as for men (IAAF Women's Committee, 1995). In response, the IAAF Congress agreed to a plan in 1995 which would see women's pole vault and hammer throw debut at the 1999 World Championships in Athletics (IAAF, 1995). Adding steeplechase races for women to IAAF events proved even more cumbersome and did not materialize until the 2005 World Athletics Championships (IAAF Women's Committee, 2002). However, it was only in 2017 that the women's competition programme reached the same number of events as the men (Krech, 2019).

Regarding development work for women's athletics, the Women's Committee proposed a Strategy for the Development of Athletics for Women in 1991, which focused on detailing “the situation of women's athletics in the world” and proposing specific strategies to encourage women's involvement in all roles in the sport (IAAF Women's Committee, 1991). Such development work was to be undertaken in both “advanced” and “less advanced” athletics nations, although the strategies would differ by context (Ibid.). These goals were primarily pursued through the staging of seminars and workshops around the world. These events failed to have a sustainable impact so the Women's Committee proposed the establishment of an IAAF Year of Women in Athletics, which would involve a range of promotional activities around the world (Ibid.). This was agreed in 1995 and the Year of Women's Athletics eventually took actually place in 1998. However, the Women's Committee was denied its own budget for developing women's athletics, while its proposals were ignored in the activities of the IAAF's Regional Development Centres (RDCs), located around the world. The Women's Committee also failed to make the establishment of a women's committee in each member federation a common standard. Therefore, the historical account described lends to the reality that women tend to be underrepresented in the national federations (Anthonj et al., 2013).

More recently, the IAAF has become increasingly aware about the federation's gender inequalities and has addressed the issue of gender in its latest governance reform process to ensure that more women are represented at all levels in the sport's governance. This was primarily done through a change in the IAAF constitution to reach better gender balance on the IAAF Council, the IAAF's executive body. Several milestones were introduced that lead to 50% gender distribution in the IAAF Council and amongst the IAAF vice-presidents by 2027 (World Athletics, 2016). In 2019, the IAAF introduced a Gender Leadership Taskforce to intensify the development of specific programmes to educate potential candidates for executive roles from national federations. Significantly, the governance reform only focused on the level of representation, with issues of women's overall participation in athletics, technical aspects and global development of women's athletics still overseen by the IAAF Women's Commission.

Despite those latest changes on the governance level, it seems fair to conclude that for most of IAAF's existence, women's athletics was not an organizational top priority. The Women's Committee figured particularly low on the organizational hierarchy and its policy initiatives regularly encountered pushback from within the IAAF structure. The ignorance for the issues of women's athletics stands in stark contrast to IAAF's general efforts to diffuse athletics worldwide (Krieger, 2019). In 1976, the organization created an IAAF Development Aid Programme in order to promote the spread of athletics in particular in developing countries (Connor and McEwen, 2011). Beginning in 1985, the IAAF further established Regional Development Centers (RDCs) in developing countries. The first RDC was located in India, others followed. Moreover, the IAAF founded the International Athletics Foundation, which aims to develop and spread scientific knowledge about coaching and training, to financially help building sporting facilities and also to encourage their member states to organize competitions (World Athletics, 2012). As in other international sport governing bodies, these development policies also served the goals of the leadership of IAAF to secure votes from the benefitting countries (Krieger, 2021).

The IAAF has also increasingly pursued a decentralization strategy reflecting concerns about the commercial future of athletics. Hence, the IAAF's marketing plan of 2006 strongly suggested to better develop the African market because European markets saw decreasing audience figures and lacked star athletes (International Association of Athletics Federations, 2006b). Former IAAF president Lamine Diack promoted an Athletics World Plan in 2003, which empowered the Area Associations (International Association of Athletics Federations, 2009). Therefore, in 2008 the IAAF changed its rules for sanctioning competitions (International Association of Athletics Federations, 2008). Previously, the IAAF Council had the exclusive right to determine whether member federations could stage IAAF events (International Association of Athletics Federations, 2006a). From 2009 on, the authority was given to the six Area Associations. As a result, all six Area Associations held events in the second highest competition category, called World Challenge, in 2010 for the first time. In addition, the IAAF lowered the performance requirements for athletes to appear in the season's best list. In short, the IAAF decentralized its competition programs to increase visibility for more member federations, enhance its marketing opportunities and promote the development of athletics.

In summary, previous research has shown that the development of women's athletics has faced multiple challenges, which included opposition from men's officials in international athletics to highly unequal national gender regimes. As a result, the promotion of women's athletics was difficult. Therefore, the current study addresses two key questions:

1. How does macro-social gender inequality relate to country participation in international women's athletics?
2. How did the IAAF's decentralization strategy affect the participation in international women's athletics?

## Research Design

### Data Sources

Research presented here analyzes data on season's bests in international athletics in the period from 2001 to 2019. The performance data analyzed here have been exclusively retrieved from the official website of World Athletics (formerly IAAF website). World Athletics is collecting the results of every performance at an officially licensed events and makes them publicly available. At the end of each year, these results are combined into season's bests lists with only the best result of an athlete in a discipline in a respective year. World Athletics allows for non-commerical use of the data as long as the data source is mentioned. Moreover, it should be mentioned the season's bests data are here only analyzed in anonymized from, that is, without considering the identity of the individual athlete. World Athletics has defined minimum performances to enter the season's best list (i.e., 11.00 s in the men's 100 m run), so that the list entries are limited. We decided to exclude the combined events (heptathlon and decathlon) from our datasets since only few countries in the world are participating here due to technical and infrastructural reasons.

Analyzing season's bests comes with a number of methodological advantages. First, in comparison to analyzing Olympic medal shares, data on season's bests are by definition available on an annual base and not only in 4-year intervals. Second, season's bests might also more accurately reflect the proficiency level of athletes and elite sport systems, as Olympic performances are heavily day dependent with athletes employing different tactics (Lames, 2002). Third, the analysis of season's bests avoids modeling problems resulting from the two-stage character of Olympic competitions (Johnson and Ali, 2004).

### Dependent Variables

The account presented here analyzes four different indicators for country participation in women's international athletics. First, we calculated the share of women's athletes in the total number of athletes of a country c in a certain discipline j and a certain year t (PARITY_c, j, t_). PARITY ranges from “0” in cases where only men's athletes participated in a discipline to “1” in cases where only women athletes participated. This serves as an indicator for the development of women's participation with respect to men's participation. In addition, two count variables were conducted for measuring the visibility of member federations in women's athletics, that is, the number of women elite athletes per 100,000 inhabitants appearing in the season's best lists in a certain discipline j for a country c in a certain year t and the number of women's events per 100,000 inhabitants in a certain discipline j a country c has been hosting in a certain year t. The latter is drawn from the season's best lists' additional information about the venues where the respective result has been achieved. Only events licensed by World Athletics, that is, events fulfilling minimum infrastructure and participation standards appear in the season's best lists. Both variables appeared to be extremely strongly overdispersed, with more than 80 percent of the observations equaling zero. The research team decided to convert them into categorical variables with three categories, having countries with zero athletes or events in category 1, countries with up to 0.1 athletes (ATHLETES_c, j, t_) or events (HOSTINGS_c, j, t_) per 100,000 inhabitants in category 2 and all with more than 0.1 in category 3. Finally, the number of athletic disciplines in which a particular country c participated in a certain year t was counted (DISCIPLINES_c, t_). This dependent variable serves as an indicator for a countries visibility in athletics in general.

### Independent Variables

As discussed above, previous scholarship has used quite different indicators for gender (in)equality in the public sphere. After intense discussion, the women's political empowerment index (WPEI) as developed by the V-Dem Institute was chosen as indicator because it seems to allow for more precise measurement and covers the Global South better than other indices. The V-dem Institute offers free access to datasets with democratic indicators for 202 countries over a period from 1789 to 2020 (Coppedge et al., 2021). The WPEI, as one of these indicators, considers three dimensions of empowerment, that is, women's civil liberties, civil society participation and political participation and originally ranges between “0” (no political empowerment) and “1” (full political empowerment) (Sundström et al., 2017). For the analyses presented here, a categorical variable with five categories (from “1 = very low WPEI” to “5 = very high WPEI”) was created. Hence, with regard to the research questions, WPEI represents the first key independent variable. The second key independent variable is a set of year dummies for the period from 2001 to 2019 in order to estimate a potential effect of World Athletics' strategy change (YEAR). The year dummies do not only allow to estimate the effects of the decentralization strategy of World Athletics but also to account for general trends.

### Controls

As religion seems to play an important role for women's sporting participation and women's success (Sfeir, 1985; Trivedi and Zimmer, 2014; Noland and Stahler, 2016), a categorical variable for RELIGION was created based on a country's majority religion. Data on the religious affiliation of a country's population was retrieved from the Pew Research Center website (Pew Research Center, 2015). Since the IAAF developed its decentralization strategy in particular to promote the diffusion of athletics in Africa, the second control variable categorizes World Athletics' distinct Area Associations (ASSOCIATION). Moreover, the existence of a national elite sport tradition was considered by measuring the age of the first acknowledged National Olympic Committee (NOC) (NOCAGE). Since the literature on the specialization of national elite sport systems assumed that countries with lower resource endowments are more prone to make strategic choices, the analyses control for the strength of the national economy (GDP PER CAP) and country size (POPULATION) by including two categorical variables. POPULATION and GDP PER CAP were retrieved from the World Development Indicator (WDI) database as provided by the World Bank (World Bank, 2020). In order to account for differences among athletic disciplines, they were combined into groups (DISCIPLINE GROUP) (Table 1).

**Table 1:** Dependent and independent variables for all regression models.

**Name**	**Definition**	**Level**	**Type**	**Obs**	**Min**	**Max**	**Mean**	**SD**
**Dependent variables**								
PARITY	Share of women's athletes a country has in a discipline per year	Country	Continuous	79,580	0	1	0.202	0.300
ATHLETES		Country	Categorical					
No women's athletes	No women's athletes of a country appear in a discipline in a year			45,996	0	1	0.615	0.487
<0.1 women's athletes	Between 0.001 and 0.1 women's athletes per 100,000 inhabitants in a discipline in a year			21,739	0	1	0.290	0.454
≥0.1 women's athletes	More than 0.1 women's athletes per 100,000 inhabitants in a discipline in a year			7,112	0	1	0.095	0.293
HOSTINGS		Country	Categorical					
No hostings	No events for women's athletes in a country in a discipline in a year			51,276	0	1	0.685	0.465
<0.1 hostings	Between 0.001 and 0.1 events per 100,000 inhabitants for women's athletes in a discipline in a year			20,089	0	1	0.268	0.443
≥0.1 hostings	More than 0.1 events per 100,000 inhabitants for women's athletes in a discipline in a year			3,514	0	1	0.047	0.211
DISCIPLINES	Number of disciplines in which a country participates per year.	Country	Continuous	3,857	0	20	7.610	7.532
**Independent variables**								
WPEI		Country	Categorical					
Very low WPEI	Countries with a WPEI lower than 0.565			13,360	0.058	0.565	0.416	0.106
Low WPEI	Countries with a WPEI between 0.566 and 0.722			13,460	0.567	0.722	0.654	0.044
Middle WPEI	Countries with a WPEI between 0.723 and 0.819			13,440	0.723	0.819	0.776	0.029
High WPEI	Countries with a WPEI between 0.820 and 0.887			13,180	0.820	0.887	0.856	0.020
Very high WPEI	Countries with a WPEI between 0.888 and 0.969			13,280	0.888	0.969	0.928	0.021
YEAR		Country	Categorical					
2001	1 = Observation is from 2001; otherwise = 0			4,220	0	1	0.053	0.224
2002	1 = Observation is from 2001; otherwise = 0			4,180	0	1	0.053	0.223
2003	1 = Observation is from 2001; otherwise = 0			4,180	0	1	0.053	0.223
2004	1 = Observation is from 2001; otherwise = 0			4,180	0	1	0.053	0.223
2005	1 = Observation is from 2001; otherwise = 0			4,180	0	1	0.053	0.223
2006	1 = Observation is from 2001; otherwise = 0			4,220	0	1	0.053	0.224
2007	1 = Observation is from 2001; otherwise = 0			4,180	0	1	0.053	0.223
2008	1 = Observation is from 2001; otherwise = 0			4,180	0	1	0.053	0.223
2009	1 = Observation is from 2001; otherwise = 0			4,180	0	1	0.053	0.223
2010	1 = Observation is from 2001; otherwise = 0			4,180	0	1	0.053	0.223
2011	1 = Observation is from 2001; otherwise = 0			4,220	0	1	0.053	0.224
2012	1 = Observation is from 2001; otherwise = 0			4,180	0	1	0.053	0.223
2013	1 = Observation is from 2001; otherwise = 0			4,180	0	1	0.053	0.223
2014	1 = Observation is from 2001; otherwise = 0			4,220	0	1	0.053	0.224
2015	1 = Observation is from 2001; otherwise = 0			4,180	0	1	0.053	0.223
2016	1 = Observation is from 2001; otherwise = 0			4,180	0	1	0.053	0.223
2017	1 = Observation is from 2001; otherwise = 0			4,180	0	1	0.053	0.223
2018	1 = Observation is from 2001; otherwise = 0			4,180	0	1	0.053	0.223
2019	1 = Observation is from 2001; otherwise = 0			4,180	0	1	0.053	0.223
RELIGION		Country	Categorical					
Buddhism	At least 50% of the population are Buddhists			2,660	0	1	0.033	0.179
Christianity	At least 50% of the population are Christians			50,700	0	1	0.631	0.483
Hinduism	At least 50% of the population are Hindus			1,140	0	1	0.014	0.118
Islam	At least 50% of the population are Muslims			18,620	0	1	0.232	0.422
No religion	At least 50% of the population have no religion			2,280	0	1	0.028	0.166
Other	Countries with other religions then the afore mentioned			4,940	0	1	0.061	0.166
POPULATION		Country	Categorical					
Very small population	<1 m. inhabitants			16,920	0	1	0.211	0.408
Small population	1–5 m. inhabitants			15,480	0	1	0.193	0.395
Low middle population	5–50 m. inhabitants			37,200	0	1	0.464	0.499
Middle population	50–100 m. inhabitants			5,820	0	1	0.073	0.259
Big population	>100 m. inhabitants			4,760	0	1	0.059	0.236
GDP PER CAPITA		Country	Categorical					
Low income	<995 USD			17,000	0	1	0.212	0.408
Middle income	995 to 3,895 USD			20,260	0	1	0.252	0.434
Upper middle income	3,895 to 12,055 USD			19,040	0	1	0.237	0.425
High income	> 12,055 USD			24,040	0	1	0.299	0.458
NOCAGE	Number of years since a country's National Olympic Committee has been officially recognized by the IOC	Country	Continuous	77,220	0	125	51.708	29.525
ASSOCIATION		Country	Categorical					
Africa	1 = Country belongs to Area Association of Africa, otherwise = 0			20,900	0	1	0.260	0.439
Asia	1 = Country belongs to Area Association of Asia, otherwise = 0			18,620	0	1	0.232	0.422
ConSudAtle	1 = Country belongs to Area Association of South America, otherwise = 0			4,940	0	1	0.061	0.240
Europe	1 = Country belongs to Area Association of Europe, otherwise = 0			19,760	0	1	0.248	0.432
NACAC	1 = Country belongs to Area Association of North America, otherwise = 0			10,640	0	1	0.132	0.339
Oceania	1 = Country belongs to Area Association of Oceania, otherwise = 0			5,320	0	1	0.061	0.249
DISCIPLINE GROUP		Discipline	Categorical					
Sprint	1 = Discipline is 100, 200, or 400 m, otherwise = 0			12,051	0	1	0.150	0.357
Middle distance running	1 = Discipline is 800 or 1,500 m, otherwise = 0			8,034	0	1	0.100	0.300
Long distance running	1 = Discipline is from 3,000 m up to Marathon, otherwise = 0			12,051	0	1	0.150	0.357
Hurdles and steeple chase	1 = Discipline is 100 or 110 m Hurdles, 400 m Hurdles or 3,000 m Steeplechase, otherwise = 0			12,051	0	1	0.150	0.357
Jumping	1 = Discipline is long jump, high jump, triple jump or pole vault, otherwise = 0			16,068	0	1	0.200	0.400
Throwing	1 = Discipline is javelin throw, discus throw, hammer throw or shot put, otherwise = 0			16,068	0	1	0.200	0.400
Walk	1 = Discipline is 20 or 50 km walk, otherwise = 0			4,017	0	1	0.050	0.218

### Analytic Strategy

The research questions are first addressed with some descriptive analyses of the key indicators. For conducting multivariate analyses, two different data sets were created:

The so-called “Participation dataset” contains 79,580 observations for each of the 20 disciplines for 210 countries in a certain year. It entails the dependent variables PARITY, ATHLETES, and HOSTINGS as well as the independent and control variables. Since PARITY appeared to be nearly normally distributed, ordinary least square (OLS) regressions were employed.

For ATHLETES and HOSTINGS we employed ordered logistic regressions. In all models we includes country dummies^1^ to account for the fixed effects-panel shape of the data and year dummies to map developments over the years.

The “Discipline dataset” is country based and contains 3,857 observations on country level with the dependent variable DISCIPLINES. The analyses employ tobit panel regressions for censored data since the number of disciplines for women is limited to 20. Only fixed effects models were calculated by including country dummies. Again, including year dummies serves to account for the longitudinal character of the data. The dataset includes all independent and control variables, except for DISCIPLINE GROUP.

## Findings

### Descriptive Findings

**Figure 1:**
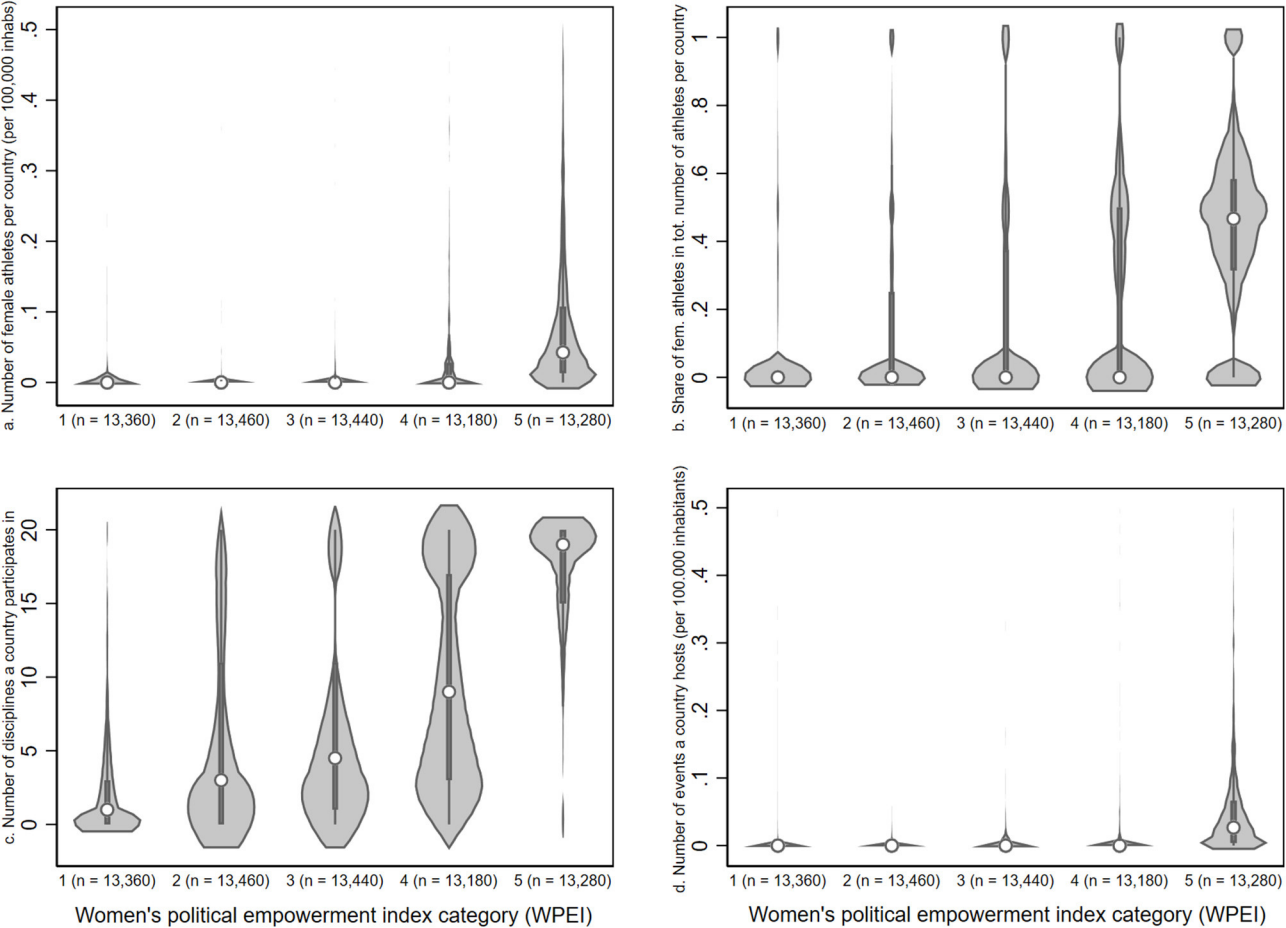
Gender (in)equality and participation in women's athletics. The figure displays violin graphs for different dependent variables and five categories of the women's political empowerment index (WPEI); 1 = low empowerment; 5 = high empowerment.

With regard to the relationship between gender (in)equality and country participation in international women's sport, Figure 1 demonstrates that in countries with more political empowerment of women, the share of women's athletes, the number of women's athletes, as well as the number of women's disciplines in which a country makes visible appearances tend to be higher. Moreover, countries with more macro-social gender equality seem to host more women's events (Figure 1).

**Figure 2:**
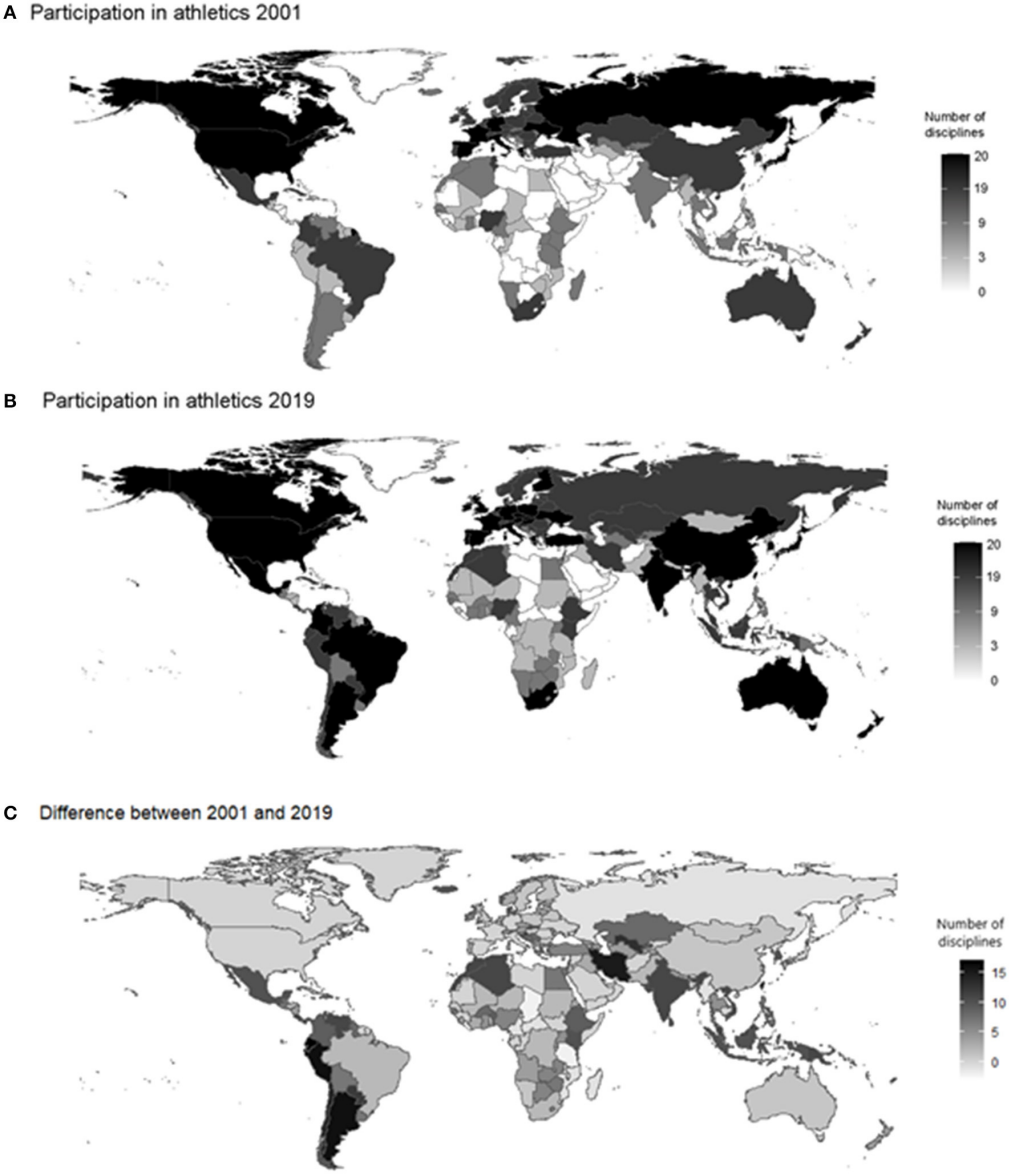
Country participation in women's athletics in 2001 and 2019 and difference between 2001 and 2019. **(A,B)** Displayed are the 5 quantiles of the number of women's athletics disciplines in which athletes from a particular country participate from white = 0 disciplines to black = 20 disciplines. **(C)** Displayed is the difference in absolute numbers of disciplines from white = −5 to 0 disciplines to black = more than 15 disciplines.

A simple mapping of country participation patterns, which is measured by number of athletic disciplines in which women's athletes make an appearance in seasons' bests, illustrates that women's athletics has made substantial progress between 2000 and 2019. The number of “white spots” (lowest quantile = 0 disciplines) for women's athletics on the world map has substantially decreased and a number of countries has expanded its visibility in women's athletics. This is particularly evident in the third figure, which shows the differences between 2001 and 2009. The highest growth were recorded in South America and in the Islamic Republic Iran (Figure 2).

A more detailed look at the top-ten increases in terms of disciplines confirms these surprising insights. A number of South American countries heavily increased their visibility in women's athletics. The same applies to the Islamic Republic Iran. Moreover, a number of European countries also appear on the list with the highest increases in terms of visible participation in disciplines (Table 2).

**Table 2:** Top-ten countries with regard to participation increases.

**Rank**	**Nation**	**Area association**	**Disciplines 2001**	**Disciplines 2019**
1	Ecuador	ConSudAtle	2	19
2	Argentina	ConSudAtle	4	20
3	Puerto Rico	NACAC	4	20
4	Peru	ConSudAtle	1	17
5	Chinese Taipeh	Asia	5	20
6	Luxembourg	Europe	0	15
7	Islamic Republic Iran	Asia	0	15
8	Croatia	Europe	6	20
9	Chile	ConSudAtle	4	18
10	Cyprus	Europe	6	19

*Displayed is the number of women's athletics disciplines in which athletes from a particular country participate*.

**Figure 3:**
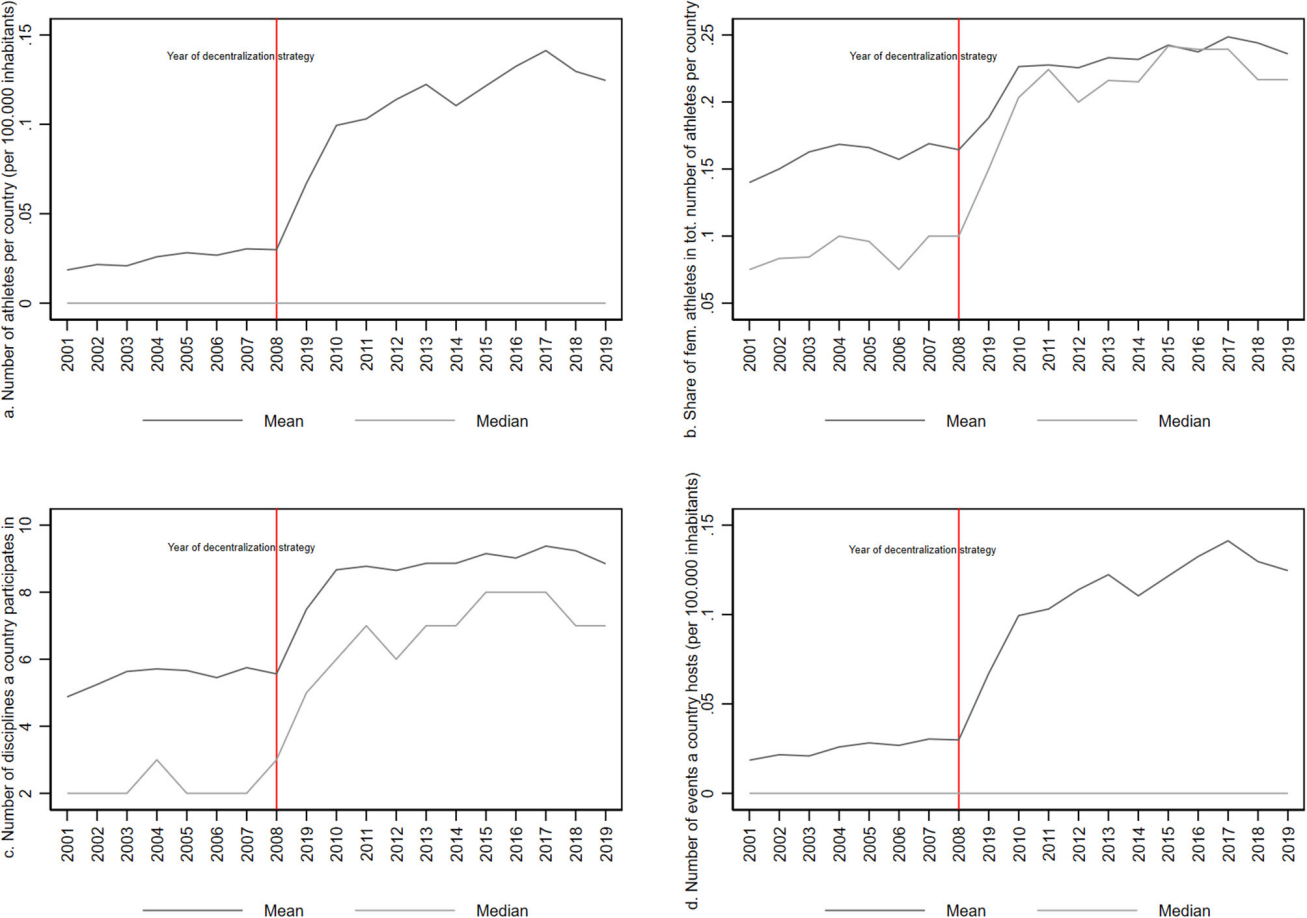
Development of participation in women's athletes between 2001 and 2019. The figure displays trends for different dependent variables.

Figure 3 suggests that the progress of women's athletics between 2001 and 2019 is related to IAAF's decentralization strategy. Hence, after the implementation of the decentralization strategy substantial increases materialized in the average share of women's athletes, the average number of women's athletes, the average number of hosted events as well as the average number of disciplines in which countries make appearances. However, as the depiction of medians makes evident, the majority of countries have neither women athletes nor events in women athletics (Figure 3).

### Multivariate Analyses

Separate multivariate analyses are conducted for the distinct dependent variables. Since PARITY ranges between 0 and 1 and is nearly normally distributed, OLS regressions were calculated. We included country dummies to account for fixed effects and year dummies to account for time-dependent developments and effects of IAAF's decentralization strategy in 2008. Two different models are presented: Model 1a represents the basic model, whereas in model 1b interactions between YEAR and WPEI were included (Table 3). Both models appear to fit the data quite well with an adjusted *R*^2^ of 0.365 or 0.367, respectively, but still leave a fairly high proportion of unexplained variance. In addition, the coefficients appear to be very stable in both models.

**Table 3:** OLS regression models for parity.

**Independent variables**	**Model 1a^a^**	**Model 1b^a^**
**WPEI^b^**		
Low WPEI	−0.012^†^ (0.007)	−0.008 (0.013)
Middle WPEI	−0.012 (0.008)	0.043** (0.015)
High WPEI	−0.003 (0.010)	−0.037* (0.017)
Very high WPEI	0.023† (0.012)	−0.014 (0.018)
**YEAR^c^**		
2002	0.006 (0.007)	0.006 (0.012)
2003	0.015† (0.008)	0.008 (0.013)
2004	0.012 (0.010)	0.014 (0.015)
2005	0.001 (0.012)	0.007 (0.016)
2006	−0.018 (0.014)	−0.002 (0.018)
2007	−0.012 (0.017)	0.001 (0.020)
2008	−0.024 (0.019)	−0.004 (0.022)
2009	−0.004 (0.022)	0.015 (0.025)
2010	0.029 (0.025)	0.019 (0.027)
2011	0.023 (0.027)	0.019 (0.029)
2012	0.016 (0.030)	0.007 (0.032)
2013	0.015 (0.032)	0.015 (0.034)
2014	0.009 (0.035)	0.007 (0.036)
2015	0.013 (0.038)	0.030 (0.039)
2016	0.002 (0.040)	0.015 (0.042)
2017	0.005 (0.043)	0.005 (0.044)
2018	−0.010 (0.046)	−0.010 (0.047)
2019	−0.022 (0.048)	−0.002 (0.050)
**RELIGION^d^**		
Buddhism	−0.141*** (0.018)	−0.135*** (0.018)
Hinduism	−0.334*** (0.039)	−0.363*** (0.039)
Islam	−0.568*** (0.047)	−0.519*** (0.048)
No religion	−0.286*** (0.027)	−0.286*** (0.027)
Other	−0.053 (0.045)	−0.017 (0.045)
**POPULATION^e^**		
Small population	0.035* (0.015)	0.045** (0.015)
Low middle population	0.041* (0.020)	0.062** (0.020)
Middle population	0.027 (0.024)	0.048* (0.024)
Big population	0.092 (0.031)	0.109** (0.032)
**GDP PER CAPITA^f^**		
Middle income	−0.002 (0.005)	0.003 (0.005)
Upper middle income	0.011 (0.007)	0.013^†^ (0.007)
High income	0.024* (0.010)	0.020* (0.010)
NOCAGE	0.007* (0.003)	0.004 (0.003)
**ASSOCIATION^g^**		
Africa	−0.476*** (0.040)	
Asia	−0.331** (0.116)	
ConSudAtle	−0.550*** (0.157)	
NACAC	−0.782** (0.267)	
Oceania	−0.504*** (0.032)	
**DISCIPLINE GROUP^h^**		
Sprint	0.122*** (0.005)	0.122*** (0.005)
Middle distance running	0.115*** (0.005)	0.115*** (0.005)
Long distance running	0.055*** (0.005)	0.055*** (0.005)
Hurdles and Steeple chase	0.099*** (0.005)	0.099*** (0.005)
Jumping	0.086*** (0.005)	0.086*** (0.005)
Throwing	0.102*** (0.005)	0.102*** (0.005)
**INTERACTIONS^i^**		
Middle WPEI × 2010		0.076*** (0.019)
Middle WPEI × 2011		0.081*** (0.019)
Middle WPEI × 2012		0.095*** (0.019)
Middle WPEI × 2013		0.076*** (0.019)
Middle WPEI × 2014		0.069*** (0.020)
Middle WPEI × 2015		0.053** (0.019)
Middle WPEI × 2016		0.056** (0.019)
Middle WPEI × 2017		0.061** (0.019)
Middle WPEI × 2018		0.066** (0.019)
Middle WPEI × 2019		0.034^†^ (0.020)
High WPEI × 2009		0.035^†^ (0.020)
High WPEI × 2010		0.054** (0.019)
High WPEI × 2011		0.052** (0.019)
High WPEI × 2012		0.047* (0.019)
High WPEI × 2014		0.051** (0.019)
High WPEI × 2015		0.043* (0.019)
High WPEI × 2017		0.070*** (0.020)
High WPEI × 2018		0.060** (0.020)
High WPEI × 2019		0.049* (0.020)
Very high WPEI × 2012		0.052** (0.019)
Very high WPEI × 2016		0.041* (0.019)
Very high WPEI × 2017		0.057** (0.019)
Very high WPEI × 2018		0.070*** (0.019)
Very high WPEI × 2019		0.053** (0.020)
Constant	0.274*** (0.033)	0.289*** (0.034)
Number of observations	65,880	65,880
*R* ^2^	0.367	0.369
Adj. *R*^2^	0.365	0.367

*Dependent variable is PARITY; Method is ordinary least squares regression with country dummies to account for the fixed effects-character of the data. Coefficients for country dummies are not reported*.

a *Displayed are regression coefficients (standard errors in bracket).*

b *Reference category is “Very low WPEI.”*

c *Reference category is “2001.”*

d *Reference category is “Christianity.”*

e *Reference category is “Very small population.”*

f *Reference category is “Very low income.”*

g *Reference category is “Europe.”*

h *Reference category is “Walk.”*

i *Reference category is “Very low WPEI × YEAR.” Only significant interaction coefficients are reported. For all coefficients see Appendix (Table A6).*

*** *p < 0.001*,

** *p < 0.01*,

* *p < 0.05*,

† *p < 0.1*.

First of all, the results do not confirm the descriptive findings as clearly as we expected: IAAF's decentralization strategy in 2008 did not significantly increase the share of women's athletes for all nations substantially since there no significant coefficients for the YEAR dummies after 2008. A higher WPEI correlates only slightly with higher share of women's athletes. PARITY is substantially higher in Christian countries than in countries with other dominant religious affilitations (RELIGIONS). Europe, compared to the other Associations, has the highest women's athlete share (ASSOCIATION), indicated by the highly significant, negative coefficients for all other associations. Interestingly, a higher share of women's athletes is found in countries with small or low middle populations (POPULATION), with a higher GDP per capita and in those with longer sporting traditions (NOCAGE) (Model 1a). The interaction coefficients in model 1b indicate that the STRATEGY CHANGE has served to increase women's athlete share in particular among countries in the middle WPEI categories. There are also discipline specific differences: Sprint, middle distance running and throwing seem to be the most equal discipline groups, especially compared to walking, which is the reference category.

For analyzing ATHLETES, which is a categorical variable, ordered logistic regressions were employed. Again, a basic (model 2a) and an interaction model (model 2b) were calculated. Model 2a does again not show a significant effect of IAAF's decentralization strategy (YEAR). WPEI and RELIGION have no significant impact on ATHLETES while countries with low middle population (POPULATION) and middle incomes (GDP PER CAPITA) seem to be more likely to have women's athletes appearing in the season's bests. Additionally, there are no significant differences among the Associations (ASSOCIATION). The interaction model provides a more nuanced view: IAAF's decentralization strategy served primarily to increase the likelihood of countries with a higher WPEI to make a visible appearance in women's international athletics over the entire period under scrutiny (Table 4). Including the interaction terms slightly served to increase the model fit, indicated by the decreased AIC. In order to check for rubustness, we calculated the basic model again for each of the different categories of WPEI (see Appendix, Table A6). The results in general confirm the original findings and offer even more insights: Again we see that higher WPEI countries increased their number of women athletes after 2008. Additionally, we find countries with low WPEI (WPEI = 2) also appear to have increased their participation after the decentralization strategy was implemented. There is a significant effect for Muslim countries. In general, the wide variation in AICs suggests that the macro-social models employed fail to account for adequately for country specific features beyond WPEI.

**Table 4:** Ordered logistic regression models for Athletes.

**Independent variables**	**Model 2a^a^**	**Model 2b^a^**
**WPEI^b^**		
Low WPEI	1.142 (0.115)	1.967** (0.463)
Middle WPEI	0.855 (0.098)	1.534^†^ (0.382)
High WPEI	0.777* (0.098)	1.197 (0.304)
Very high WPEI	0.998 (0.144)	0.991 (0.252)
**YEAR^c^**		
2002	2.854 (2.967)	5.011 (5.436)
2003	8.117 (16.844)	19.815 (41.988)
2004	20.178 (62.789)	85.520 (270.645)
2005	44.663 (185.292)	294.876 (1,242.444)
2006	91.941 (476.980)	836.368 (4402.206)
2007	254.938 (1586.298)	2,903.054 (18329.790)
2008	551.246 (4001.603)	9,624.344 (70878.970)
2009	3,966.807 (3.291 × 10^4^)	6.430 × 10^4^ (5.411 × 10^5^)
2010	18,874.960 (1.762 × 10^5^)	2.596 × 10^5^ (2.457 × 10^6^)
2011	45,952.360 (4.765 × 10^5^)	1.016 × 10^6^ (1.070 × 10^7^)
2012	1.079 × 10^5^ (1.231 × 10^6^)	2.921 × 10^6^ (3.380 × 10^7^)
2013	2.680 × 10^5^ (3.336 × 10^6^)	9.995 × 10^6^ (1.260 × 10^8^)
2014	6.818 × 10^5^ (9.192 × 10^6^)	3.490 × 10^7^ (4.780 × 10^8^)
2015	1.730 × 10^6^ (2.510 × 10^7^)	1.540 × 10^7^ (2.270 × 10^9^)
2016	4.127 × 10^6^ (6.420 × 10^7^)	5.000 × 10^8^ (7.890 × 10^9^)
2017	1.060 × 10^7^ (1.750 × 10^8^)	1.410 × 10^9^ (2.370 × 10^10^)
2018	2.330 × 10^7^ (4.110 × 10^8^)	3.680 × 10^9^ (6.580 × 10^10^)
2019	4.370 × 10^7^ (8.160 × 10^8^)	1.370 × 10^10^ (2.590 × 10^11^)
**RELIGION^d^**		
Buddhism	2.385 (2.502)	2.538 (2.698)
Hinduism	0.000 (0.000)	0.000 (0.000)
Islam	0.000 (0.036)	0.007 (10.068)
No religion	0.000 (7.36 × 10^−4^)	2.100 × 10^−5^ (1.324 × 10^−4^)
Other	1.835E+05 (2.855 × 10^6^)	2.440 × 10^7^ (3.860 × 10^8^)
**POPULATION^e^**		
Small population	1.493^†^ (0.337)	2.099** (0.486)
Low middle population	3.084*** (0.895)	3.555*** (1.057)
Middle population	0.979 (0.312)	1.218 (0.397)
Big population	0.662 (0.244)	1.053 (0.398)
**GDP PER CAPITA^f^**		
Middle income	0.619*** (0.039)	0.899 (0.060)
Upper middle income	0.533*** (0.046)	1.031 (0.0961)
High income	0.748* (0.085)	1.251 (0.147)
NOCAGE	0.430 (0.445)	0.311 (0.327)
**ASSOCIATION^g^**		
Africa	1.140.164 (15371.410)	1.077 × 10^5^ (1.473 × 10^6^)
Asia	1.340 × 10^15^ (5.830 × 10^16^)	1.000 × 10^21^ (4.430 × 10^22^)
ConSudAtle	7.250 × 10^20^ (4.360 × 10^22^)	7.080 × 10^28^ (4.320 × 10^30^)
NACAC	4.630 × 10^37^ (4.750 × 10^39^)	2.380 × 10^51^ (2.480 × 10^53^)
Oceania	0.127 (0.796)	1.491 (9.479)
**DISCIPLINE GROUP^h^**		
Sprint	14.629*** (0.910)	1.482*** (0.926)
Middle distance running	8.375*** (0.540)	8.405*** (0.545)
Long distance running	3.078*** (0.192)	3.059*** (0.191)
Hurdles and Steeple chase	4.958*** (0.307)	4.948*** (0.307)
Jumping	4.898*** (0.296)	4.885*** (0.296)
Throwing	4.473*** (0.270)	4.454*** (0.270)
**INTERACTIONS^i^**		
Low WPEI × 2004		0.600^†^ (0.177)
Low WPEI × 2005		0.499* (0.147)
Low WPEI × 2006		0.462* (0.139)
Low WPEI × 2007		0.515* (0.158)
Low WPEI × 2008		0.446** (0.136)
Low WPEI × 2009		0.500* (0.144)
Low WPEI × 2011		0.524* (0.149)
Low WPEI × 2012		0.516* (0.149)
Low WPEI × 2014		0.605^†^ (0.170)
Low WPEI × 2015		0.449** (0.128)
Low WPEI × 2016		0.407** (0.116)
Low WPEI × 2017		0.612^†^ (0.176)
Low WPEI × 2019		0.506* (0.147)
Middle WPEI × 2004		0.570^†^ (0.175)
Middle WPEI × 2006		0.582^†^ (0.178)
Middle WPEI × 2009		0.594^†^ (0.171)
Middle WPEI × 2014		0.721^†^ (0.207)
Middle WPEI × 2015		0.583^†^ (0.163)
Middle WPEI × 2019		0.565* (0.164)
High WPEI × 2005		0.537* (0.161)
High WPEI × 2006		0.493* (0.150)
High WPEI × 2008		0.471* (0.144)
Very high WPEI × 2004		0.484* (0.140)
Very high WPEI × 2005		0.422** (0.121)
Very high WPEI × 2006		0.471** (0.135)
Very high WPEI × 2007		0.434** (0.127)
Very high WPEI × 2008		0.401** (0.117)
Very high WPEI × 2010		2.177** (0.593)
Very high WPEI × 2011		1.720* (0.468)
Very high WPEI × 2012		2.388** (0.654)
Very high WPEI × 2013		2.053** (0.554)
Very high WPEI × 2014		2.635*** (0.713)
Very high WPEI × 2015		1.758* (0.474)
Very high WPEI × 2016		2.110** (0.564)
Very high WPEI × 2017		2.647*** (0.713)
Very high WPEI × 2018		3.360*** (0.926)
Very high WPEI × 2019		1.861* (0.516)
Cut1	−5.281 (8.304)	−7.078 (8.421)
Cut2	−1.276 (8.304)	−2.958 (8.420)
Number of observations	63,807	63,807
AIC	63132.62	62594.93
Pseudo *R*^2^	0.455	0.461

*Dependent variable is ATHLETES; Method is ordered logistic regression with country dummies to account for the fixed effects-character of the data. Coefficients for country dummies are not reported.*

a *Displayed are odds ratios (standard errors in bracket).*

b *Reference category is “Very low WPEI.”*

c *Reference category is “2001.”*

d *Reference category is “Christianity.”*

e *Reference category is “Europe.”*

f *Reference category is “Very small population.”*

g *Reference category is “Very low income.”*

h *Reference category is “Walk.”*

i *Reference category is “Very low WPEI × YEAR.” Only significant interaction coefficients are reported. For all coefficients see Appendix.*

*** *p < 0.001,*

** *p < 0.01,*

* *p < 0.05*,

† *p < 0.1*.

In order to examine whether women's or men's elite sport participation benefitted more from World Athletics' strategy change, we tested how PARITY has developed with respect to the number of women's athletes (ATHLETES). Therefore, we employed an OLS regression with PARITY as dependent variable and an interaction of ATHLETES and YEAR as independent variable (Table 5). To account for country specific differences, country dummies were included. Negative coefficients for the interactions would indicate that men's elite sport participation benefitted more from the strategy change, since the absolute number of women's athletes, as shown, has been generally increasing.

**Table 5:** Influence of the interaction between Athletics and Year on Parity.

**Independent variables**	**Parity (FE)^a^**
**ATHLETES^b^**	
<0.1 women's athletes	0.639*** (0.005)
≥0.1 women's athletes	0.713*** (0.015)
**Year^c^**	
2002	−0.001 (0.004)
2003	−0.003 (0.004)
2004	−0.002 (0.004)
2005	−0.003 (0.004)
2006	−0.003 (0.004)
2007	−0.003 (0.004)
2008	−0.003 (0.004)
2009	−0.007 (0.004)
2010	−0.009 (0.004)
2011	−0.009 (0.004)
2012	−0.009 (0.004)
2013	−0.008 (0.004)
2014	−0.009 (0.004)
2015	−0.009 (0.004)
2016	−0.009 (0.004)
2017	−0.009 (0.004)
2018	−0.010 (0.004)
2019	−0.010 (0.004)
**INTERACTIONS**	
<0.1 women's athletes × 2009	−0.078*** (0.007)
<0.1 women's athletes × 2001	−0.068*** (0.007)
<0.1 women's athletes × 2011	−0.070*** (0.007)
<0.1 women's athletes × 2012	−0.070*** (0.007)
<0.1 women's athletes × 2013	−0.062*** (0.007)
<0.1 women's athletes × 2014	−0.062*** (0.007)
<0.1 women's athletes × 2015	−0.055*** (0.007)
<0.1 women's athletes × 2016	−0.064*** (0.007)
<0.1 women's athletes × 2017	−0.066*** (0.007)
<0.1 women's athletes × 2018	−0.069*** (0.007)
<0.1 women's athletes × 2019	−0.058*** (0.007)
≥ 0.1 women's athletes × 2009	−0.117*** (0.017)
≥ 0.1 women's athletes × 2001	−0.105*** (0.016)
≥ 0.1 women's athletes × 2011	−0.110*** (0.016)
≥ 0.1 women's athletes × 2012	−0.106*** (0.016)
≥ 0.1 women's athletes × 2013	−0.113*** (0.016)
≥ 0.1 women's athletes × 2014	−0.120*** (0.016)
≥ 0.1 women's athletes × 2015	−0.115*** (0.016)
≥ 0.1 women's athletes × 2016	−0.106*** (0.016)
≥ 0.1 women's athletes × 2017	−0.100*** (0.016)
≥ 0.1 women's athletes × 2018	−0.094*** (0.016)
≥ 0.1 women's athletes × 2019	−0.088*** (0.016)
Constant	0.006 (0.007)
Number of observations	74,847
*R* ^2^	0.794
Adj *R*^2^	0.793

*Dependent variable is PARITY; Method is Ordinary least squares regression (OLS) with country dummies to account for the fixed effects-character of the data. Coefficients for country dummies are not reported.*

a *Displayed are regression coefficients (standard errors in bracket).*

b *Reference category is “No women's athletes.”*

c *Reference category is “2001.”*

*^d^Reference category is “No women's athletes × YEAR.” Only significant interaction coefficients are reported. For all coefficients see Appendix.*

*** *p < 0.001,*

^**^p < 0.01,

^*^p < 0.05,

*^†^p < 0.1*.

Actually, the results indicate that an increasing number of women's athletes per 100,000 inhabitants is negatively associated with the development of PARITY. There is a substantial and significant drop from 2008 to 2009 with respect to the number of women's athletes. Accordingly, it can be inferred that men's participation in elite athletics has developed better after the IAAF implemented its decentralization strategy. The model fits the data very well, indicated by an adjusted *R*^2^ of 0.793.

In order to analyze HOSTING, which represents also a categorical variable, again ordered logistic regressions were employed. As for ATHLETES, Model 3a does not show a significant effect of IAAF's decentralization strategy (YEAR). We see more events in countries with very high WPEI (WPEI), with big populations (POPULATION) and high income (GDP PER CAPITA). The interacted model (model 3b) is hard to interpret: the number of events seems to have increased in all WPEI categories and independently of the IAAF decentralization strategy since we see highly significant and positive odds ratios also before 2008 (Table 6). The robustness checks (see Appendix, Table A7) again confirm our general models. Additionally, we see that the number of events especially increased in countries with high and very high WPEI already before 2008. The models also provide stronger evidence that in particular countries with low WPEI seem to have increased the hosting of women's events after the decentralization strategy was implemented. Again, the variation in AICs suggest, however, that pure macro-social models do not grasp the developments very well.

**Table 6:** Ordered logistic regression models for Hosting.

**Independent variables**	**Model 3a^a^**	**Model 3b^a^**
**WPEI^b^**		
Low WPEI	1.470** (0.190)	4.380*** (1.747)
Middle WPEI	1.443* (0.208)	3.359** (1.375)
High WPEI	1.199 (0.188)	3.224** (1.329)
Very high WPEI	1.953*** (0.338)	4.039** (1.656)
**YEAR^c^**		
2002	1.401 (0.943)	2.716 (2.268)
2003	1.550 (2.076)	5.397 (7.732)
2004	1.564 (3.139)	12.463 (25.948)
2005	1.550 (4.147)	7.489 (20.644)
2006	1.374 (4.602)	20.976 (71.759)
2007	1.434 (5.751)	24.712 (101.215)
2008	1.096 (5.128)	23.435 (111.841)
2009	3.615 (19.335)	134.580 (732.908)
2010	6.191 (37.252)	181.033 (1108.468)
2011	5.871 (39.249)	275.233 (1871.690)
2012	6.509 (47.869)	267.045 (1997.054)
2013	5.720 (45.886)	259.624 (2117.575)
2014	5.950 (51.710)	237.110 (2094.792)
2015	6.893 (64.514)	934.490 (8889.398)
2016	6.403 (64.207)	512.338 (5221.275)
2017	8.080 (86.424)	648.945 (7053.800)
2018	7.717 (87.705)	743.207 (8582.922)
2019	11.056 (133.042)	2877.926 (35187.250)
**RELIGION^d^**		
Buddhism	1.373 (0.944)	1.704 (1.189)
Hinduism	0.015 (0.121)	0.001 (0.007)
Islam	0.000 (0.000)	0.000 (0.000)
No religion	0.056 (0.227)	0.015 (0.061)
Other	0.000 (0.000)	0.000 (0.002)
**POPULATION^e^**		
Small population	0.824 (0.294)	0.810 (0.292)
Low middle population	0.925 (0.365)	0.928 (0.372)
Middle population	0.524 (0.222)	0.617 (0.265)
Big population	0.370* (0.171)	0.530 (0.250)
**GDP PER CAPITA^f^**		
Middle income	0.895 (0.069)	1.039 (0.086)
Upper middle income	0.985 (0.100)	1.291* (0.145)
High income	1.552** (0.202)	2.038*** (0.278)
NOCAGE	1.007 (0.673)	0.776 (0.527)
**ASSOCIATION^g^**		
Africa	0.003 (0.022)	0.077 (0.679)
Asia	0.145 (4.071)	7.18 × 10^3^ (2.05 × 10^5^)
ConSudAtle	0.132 (5.102)	4.01 × 10^5^ (1.58 × 10^7^)
NACAC	0.930 (61.570)	9.19 × 10^10^ (6.18 × 10^12^)
Oceania	0.002 (0.010)	0.013 (0.052)
**DISCIPLINE GROUP^h^**		
Sprint	10.306*** (0.702)	10.428 (0.713)
Middle distance running	5.677*** (0.404)	5.714 (0.408)
Long distance running	2.390*** (0.164)	2.397 (0.165)
Hurdles and Steeple chase	4.174*** (0.284)	4.199 (0.287)
Jumping	5.324*** (0.353)	5.362 (0.357)
Throwing	5.570*** (0.369)	5.608 (0.374)
**INTERACTIONS^i^**		
Low WPEI × 2003		0.390^†^ (0.190)
Low WPEI × 2004		0.227** (0.106)
Low WPEI × 2006		0.175*** (0.081)
Low WPEI × 2007		0.315* (0.147)
Low WPEI × 2008		0.284** (0.136)
Low WPEI × 2009		0.126*** (0.056)
Low WPEI × 2010		0.313** (0.138)
Low WPEI × 2011		0.172*** (0.076)
Low WPEI × 2012		0.245** (0.109)
Low WPEI × 2013		0.345* (0.154)
Low WPEI × 2015		0.167*** (0.074)
Low WPEI × 2016		0.224** (0.102)
Low WPEI × 2019		0.201*** (0.090)
Middle WPEI × 2006		0.284** (0.132)
Middle WPEI × 2007		0.290** (0.136)
Middle WPEI × 2008		0.308* (0.148)
Middle WPEI × 2009		0.172*** (0.077)
Middle WPEI × 2010		0.359* (0.159)
Middle WPEI × 2011		0.273** (0.121)
Middle WPEI × 2012		0.437^†^ (0.194)
Middle WPEI × 2013		0.424^†^ (0.190)
Middle WPEI × 2015		0.215** (0.095)
High WPEI × 2004		0.298** (0.138)
High WPEI × 2006		0.213** (0.099)
High WPEI × 2007		0.242** (0.113)
High WPEI × 2008		0.218** (0.105)
High WPEI × 2009		0.255** (0.112)
High WPEI × 2010		0.269** (0.118)
High WPEI × 2011		0.219** (0.096)
High WPEI × 2012		0.404* (0.178)
High WPEI × 2013		0.381* (0.168)
High WPEI × 2015		0.192*** (0.084)
Very high WPEI × 2003		0.454^†^ (0.216)
Very high WPEI × 2004		0.258** (0.117)
Very high WPEI × 2006		0.227** (0.101)
Very high WPEI × 2007		0.222** (0.100)
Very high WPEI × 2008		0.266** (0.123)
Very high WPEI × 2009		0.204*** (0.087)
Very high WPEI × 2010		0.371* (0.159)
Very high WPEI × 2011		0.349* (0.149)
Very high WPEI × 2012		0.465^†^ (0.200)
Cut1	0.915 (0.365)	−0.258 (5.459)
Cut2	5.344 (5.365)	4.237 (5.459)
Number of observations	63,839	63,839
AIC	50743.24	50,540.58
Pseudo *R*^2^	0.493	0.497

*Dependent variable is HOSTINGS; Method is ordered logistic regression with country dummies to account for the fixed effects-character of the data. Coefficients for country dummies are not reported.*

a *Displayed are odds ratios (standard errors in bracket).*

b *Reference category is “Very low WPEI.”*

c *Reference category is “2001.”*

d *Reference category is “Christianity.”*

e *Reference category is “Very small population.”*

f *Reference category is “Very low income.”*

g *Reference category is “Europe.”*

h *Reference category is “Walk.”*

i *Reference category is “Very low WPEI × YEAR.” Only significant interaction coefficients are reported. For all coefficients see Appendix (Table A7).*

*** *p < 0.001,*

** *p < 0.01,*

* *p < 0.05*,

† *p < 0.1*.

Finally, the number of disciplines in which a country is present in the season's bests (DISCIPLINES) is analyzed as proxy for the development of a national women's elite sport system. Since the dataset has panel character with a censored dependent variable, tobit regressions were conducted. Fixed effects models, which provide more consistent estimators, were calculated (Models 4a and 4b) by including country dummies. Again, a basic model and an interaction model were estimated. Both models have a very a high model fit, in particular model 4b, which predicts 82% of the data correctly (multiple *R*^2^).

First of all, all model 4a shows highly significant and positive coefficients from 2009 onwards. Accordingly, IAAF's decentralization strategy is related to an increase of the disciplines in which women's athletes of a particular country appear in the season's bests. Additionally, DISCIPLINES is significantly higher for countries with higher WPEI's. The number of DISCIPLINES per country is higher in Europe, non-Islamic and non-Buddhist countries as well as countries with larger populations and a longer sport tradition. Interestingly, GDP PER CAPITA seems to exert a negative effect. In the interacted fixed effect model (Model 4b), the mostly insignificant interaction coefficients show that developments over time were not related to WPEI (Table 7).

**Table 7:** Tobit regression models for Disciplines.

	**Model 4a (FE)^a^**	**Model 4b (FE)^a^**
**WPEI^b^**		
Low WPEI	2.583*** (0.375)	−0.281 (0.788)
Middle WPEI	4.156*** (0.375)	−1.322 (0.873)
High WPEI	6.410*** (0.407)	−1.390 (0.885)
Very high WPEI	8.836*** (0.513)	−0.414 (0.966)
**YEAR^c^**		
2002	0.605 (0.674)	1.165 (2.655)
2003	1.140 (0.670)	1.885 (5.133)
2004	0.913 (0.668)	2.584 (7.651)
2005	0.251 (0.673)	2.466 (10.181)
2006	−0.579 (0.675)	2.473 (12.711)
2007	−0.327 (0.671)	3.092 (15.243)
2008	−0.765 (0.670)	3.089 (17.777)
2009	2.492*** (0.665)	5.091 (20.312)
2010	3.998*** (0.665)	5.975 (22.847)
2011	3.957*** (0.667)	6.610 (25.382)
2012	3.536*** (0.665)	6.556 (27.918)
2013	3.604*** (0.667)	6.719 (30.454)
2014	3.568*** (0.669)	6.932 (32.991)
2015	3.913*** (0.670)	8.130 (35.528)
2016	3.783*** (0.665)	8.710 (38.065)
2017	4.191*** (0.667)	8.234 (40.602)
2018	3.703*** (0.667)	7.825 (43.139)
2019	2.977*** (0.667)	8.352 (45.676)
**RELIGION^d^**		
Buddhism	−2.044** (0.654)	−1.612 (2.661)
Hinduism	−0.628 (0.845)	−17.224 (30.459)
Islam	−1.804*** (0.322)	−29.695 (1634.722)
No religion	0.057 (0.638)	−11.893 (15.244)
Other	1.256 (0.568)	0.191 (38.065)
**POPULATION^e^**		
Small population	4.427*** (0.417)	2.612** (0.908)
Low middle population	9.263*** (0.395)	1.263 (1.117)
Middle population	13.333*** (0.600)	−0.632 (1.291)
Big population	16.607*** (0.639)	0.001 (1.597)
**GDP PER CAPITA^f^**		
Middle income	−1.138** (0.380)	−1.248*** (0.335)
Upper middle income	−3.716*** (0.412)	−2.500*** (0.423)
High income	−6.129*** (0.454)	−2.549*** (0.527)
NOCAGE	0.050 (0.005)	−0.109 (2.537)
**ASSOCIATION^g^**		
Africa	−4.280*** (0.412)	−31.058 (1574.711)
Asia	−4.987*** (0.435)	−18.174 (1577.968)
ConSudAtle	−3.213*** (0.494)	−16.956 (1581.228)
NACAC	−3.797*** (0.450)	9.030 (2221.854)
Oceania	−1.146 (0.772)	−40.276 (1574.440)
**INTERACTIONS^h^**		
Low WPEI × 2006		−1.737^†^ (1.035)
Low WPEI × 2008		−1.742^†^ (1.051)
Middle WPEI × 2010		2.180* (1.026)
Middle WPEI × 2011		1.729^†^ (1.032)
Middle WPEI × 2012		1.861^†^ (1.041)
Middle WPEI × 2013		1.787^†^ (1.033)
Middle WPEI × 2018		1.750^†^ (1.043)
High WPEI × 2006		−1.783^†^ (1.049)
High WPEI × 2010		2.258* (1.021)
High WPEI × 2012		1.667^†^ (1.003)
High WPEI × 2013		1.800^†^ (1.001)
High WPEI × 2017		2.038* (1.033)
Very high WPEI × 2007		−2.014^†^ (1.058)
Very high WPEI × 2008		−2.490* (1.062)
Constant	−2.771** (0.800)	36.237 (1574.497)
Var(e.DISCIPLINES)	31.310 (1.006)	5.754 (0.178)
Multiple *R*^2^	0.685	0.819
LR chi^2^	3767.36	8175.41
Pseudo *R*^2^	0.202	0.438
Prob>chi^2^	0.000	0.000
Left-censored obs.	690	690
Right-censored obs.	468	468
Observations	3,290	3,290

*Dependent variable is DISCIPLINES; Method is tobit regressions with country dummies to account for the fixed effects-character of the data due to the truncated dependent variable. Coefficients for country dummies are not reported.*

a *Displayed are tobit regression coefficients (standard errors in bracket).*

b *Reference category is “Very low WPEI.”*

c *Reference category is “2001.”*

d *Reference category is “Christianity.”*

e *Reference category is “Very small population.”*

f *Reference category is “Very low income.”*

g *Reference category is “Europe.”*

h *“Very low WPEI × YEAR.” Only significant interaction coefficients are reported. For all coefficients see Appendix.*

*** *p < 0.001,*

** *p < 0.01,*

* *p < 0.05*,

† *p < 0.1*.

## Discussion

The results of our study will be first discussed in the lights of the guiding research questions, that is, (1) the relevance of macro-social gender inequality for country participation in international women's athletics, and (2) the impact of IAAF's decentralization strategy on participation in international women's athletics.

With regard to the first question, the study, which relied on a larger sample of countries and more fine-grained data, primarily confirmed previous findings. It was demonstrated once more that macro-social gender equality matters for women's sport. Higher women's empowerment in the public sphere relates to higher participation of countries in international women's athletics. It became also at least slightly evident that countries with Muslim religious affiliation appear to be in general less supportive of women's participation in international elite sports. However, there are notable exceptions, such as, the Islamic Republic of Iran (see below). Interestingly, population seems to play a less important role than in men's sports, while country participation in women's international athletics increased with higher GDP per capita.

Concerning the second questions, the study demonstrated that women's athletics made substantial progress over the last two decades, which is in some aspects related to the IAAF's decentralization strategy. The number of disciplines in which countries participate substantially expanded over the period examined. Also the number of athletes and hostings generally increased. It is most interesting that the progress of women's athletics is not related to a deliberate developmental policy of the IAAF (now World Athletics) with regard to women's athletics. The progress appears to be the outcome of a more general decentralization strategy, which involved the lowering of performance requirements for season's bests and of technical standards for hosting. The decentralization strategy allowed more countries to make visible appearances in women's athletics and served to increase women's share among national elite athletes. However, the findings also indicate that although the decentralization strategy served to increase the participation of countries in women's elite athletics, men's athletics appear to have benefitted even more.

Hence, it can be concluded that the study demonstrates the limits of such rather gender unspecific development strategies. The analyses showed that the decentralization strategy mainly promoted the development of women's athletics in countries characterized by higher levels of women's empowerment. These countries include, among others, Costa Rica, where the share of women's athletes increased after the implementation of the IAAF's decentralization stratey, the United States, which experienced a remarkable growth in women's athletes appearing in the season's bests and in hosted events, and Croatia, where the number of athletic disciplines in which women's athletes appeared in the season's bests increased. By implication, the differential impact of the decentralization strategy is likely to increase the gaps in the development of women's athletics between less and more gender equal countries. It seems reasonable to assume that the decentralization strategy allowed more gender equal countries to increase their visibility in women's international athletics because of stronger grassroots of women's athletics in these countries. Accordingly, the current study suggests that a more deliberate developmental and better resourced strategy is needed to promote women's athletics in countries characterized by lower women's empowerment. If such efforts are not made, the progress of women's athletics in these countries will depend on whether women's empowerment increases and automatically translates into better opportunities for women's elite sports. Hence, if World Athletics aims to deliberately promote women's athletics in less gender equal countries, it should create better targeted women's developmental programs. The IAAF Women's Commission made similar recommendation in the period between 1990 and 2007 but received significant pushback from leading IAAF bodies. However, it should be realized that encouraging investments in women's elite sports might not the most reasonable strategy for promoting women's sport and physical activity in such countries as it is highly questionable whether such top-down approaches result in “trickle down” effects benefitting women's participation in sport or physical activity in general (Connor and McEwen, 2011).

## Limitations

First of all, it should be realized that the current study does not allow for strong causal claims as it represents only a retrospective data analysis. In addition, the current study shares the limitations of other macro-social accounts, which usually neglect meso-level factors. It is important to realize that the analyses hinted at the existence of country specific responses to IAAF's decentralization strategy. However, a macro-social approach provides little means to dissect these responses. The relevance of meso-level factors has been indicated by the substantial progress of women's athletics in the Islamic Republic of Iran. This progress in a country with strong Muslim religious affiliation seems to reflect the efforts of the Iranian government to exploit sport in pursuit of a broad range of domestic and international policy objectives (in general: Dousti et al., 2013; for women's sport: Sadeghi et al., 2018). Hence, the progress of women's elite sport depends on priorities of national sport policies. Moreover, the relevance of path dependencies and diffusion patterns is indicated by the fact that countries with a longer sport tradition seem to show a higher participation in women's international athletics. It might be speculated that, even though the first sport men's officials heavily discriminated against women, an earlier establishment of a national sport movement served also to bring earlier up the question of women's participation or women's sport. Hence, besides national gender regimes and sport policies, sport specific trajectories seem to be relevant.

Accordingly, future analyses should try to conduct more sophisticated proxies for meso-level factors in order to improve academic understanding of the development of women's sport and to provide better guidance to sport administrators at international and national level.

## Data Availability Statement

The raw data supporting the conclusions of this article will be made available by the authors, without undue reservation.

## Author Contributions

HM and MK contributed to the conception and design of the empirical study. MK organized the database and performed the statistical analyses. HM and JK wrote the theory section and the discussion section. All authors wrote sections of the manuscript.

## Conflict of Interest

The authors declare that the research was conducted in the absence of any commercial or financial relationships that could be construed as a potential conflict of interest.

## Publisher's Note

All claims expressed in this article are solely those of the authors and do not necessarily represent those of their affiliated organizations, or those of the publisher, the editors and the reviewers. Any product that may be evaluated in this article, or claim that may be made by its manufacturer, is not guaranteed or endorsed by the publisher.

## Footnotes

^1^Coefficients for the 210 country dummies will not be reported.
