# Supplementary material for: Women in International Elite Athletics: Gender (in)equality and National Participation
Source: Front Sports Act Living. 2021 Aug 27;3:709640. doi: 10.3389/fspor.2021.709640 (PMC8429847; doi:10.3389/fspor.2021.709640)
Supplement: Supplementary file 1 [file Data_Sheet_1.docx]

# Appendix

## Table A1. Regression models for Parity

| **Independent variables** | **Model 1a^a^** | **Model 1b^a^** |
| --- | --- | --- |
| WPEI^b^ |  |  |
| Low WPEI | -0.012† (0.007) | -0.008 (0.013) |
| Middle WPEI | -0.012 (0.008) | 0.043** (0.015) |
| High WPEI | -0.003 (0.010) | -0.037* (0.017) |
| Very high WPEI | 0.023† (0.012) | -0.014 (0.018) |
| YEAR^c^ |  |  |
| 2002 | 0.006 (0.007) | 0.006 (0.012) |
| 2003 | 0.015† (0.008) | 0.008 (0.013) |
| 2004 | 0.012 (0.010) | 0.014 (0.015) |
| 2005 | 0.001 (0.012) | 0.007 (0.016) |
| 2006 | -0.018 (0.014) | -0.002 (0.018) |
| 2007 | -0.012 (0.017) | 0.001 (0.020) |
| 2008 | -0.024 (0.019) | -0.004 (0.022) |
| 2009 | -0.004 (0.022) | 0.015 (0.025) |
| 2010 | 0.029 (0.025) | 0.019 (0.027) |
| 2011 | 0.023 (0.027) | 0.019 (0.029) |
| 2012 | 0.016 (0.030) | 0.007 (0.032) |
| 2013 | 0.015 (0.032) | 0.015 (0.034) |
| 2014 | 0.009 (0.035) | 0.007 (0.036) |
| 2015 | 0.013 (0.038) | 0.030 (0.039) |
| 2016 | 0.002 (0.040) | 0.015 (0.042) |
| 2017 | 0.005 (0.043) | 0.005 (0.044) |
| 2018 | -0.010 (0.046) | -0.010 (0.047) |
| 2019 | -0.022 (0.048) | -0.002 (0.050) |
| RELIGION^d^ |  |  |
| Buddhism | -0.141*** (0.018) | -0.135*** (0.018) |
| Hinduism | -0.334*** (0.039) | -0.363*** (0.039) |
| Islam | -0.568*** (0.047) | -0.519*** (0.048) |
| No religion | -0.286*** (0.027) | -0.286*** (0.027) |
| Other | -0.053 (0.045) | -0.017 (0.045) |
| POPULATION^e^ |  |  |
| Small population | 0.035* (0.015) | 0.045** (0.015) |
| Low middle population | 0.041* (0.020) | 0.062** (0.020) |
| Middle population | 0.027 (0.024) | 0.048* (0.024) |
| Big population | 0.092 (0.031) | 0.109** (0.032) |
| GDP PER CAPITA^f^ |  |  |
| Middle income | -0.002 (0.005) | 0.003 (0.005) |
| Upper middle income | 0.011 (0.007) | 0.013^†^ (0.007) |
| High income | 0.024* (0.010) | 0.020* (0.010) |
| NOCAGE^g^ | 0.007* (0.003) | 0.004 (0.003) |
| ASSOCIATION^h^ |  |  |
| Africa | -0.476*** (0.040) |  |
| Asia | -0.331** (0.116) |  |
| ConSudAtle | -0.550*** (0.157) |  |
| NACAC | -0.782** (0.267) |  |
| Oceania | -0.504*** (0.032) |  |
| DISCIPLINE GROUP^i^ |  |  |
| Sprint | 0.122*** (0.005) | 0.122*** (0.005) |
| Middle distance running | 0.115*** (0.005) | 0.115*** (0.005) |
| Long distance running | 0.055*** (0.005) | 0.055*** (0.005) |
| Hurdles and Steeple chase | 0.099*** (0.005) | 0.099*** (0.005) |
| Jumping | 0.086*** (0.005) | 0.086*** (0.005) |
| Throwing | 0.102*** (0.005) | 0.102*** (0.005) |
| INTERACTIONS^j^ |  |  |
| Low WPEI × 2002 |  | 0.005 (0.017) |
| Low WPEI × 2003 |  | 0.018 (0.017) |
| Low WPEI × 2004 |  | 0.001 (0.017) |
| Low WPEI × 2005 |  | -0.010 (0.017) |
| Low WPEI × 2006 |  | -0.014 (0.017) |
| Low WPEI × 2007 |  | -0.007 (0.018) |
| Low WPEI × 2008 |  | -0.005 (0.018) |
| Low WPEI × 2009 |  | -0.012 (0.018) |
| Low WPEI × 2010 |  | 0.016 (0.018) |
| Low WPEI × 2011 |  | 0.003 (0.018) |
| Low WPEI × 2012 |  | 0.002 (0.019) |
| Low WPEI × 2013 |  | 0.021 (0.018) |
| Low WPEI × 2014 |  | 0.023 (0.018) |
| Low WPEI × 2015 |  | -0.010 (0.019) |
| Low WPEI × 2016 |  | -0.006 (0.019) |
| Low WPEI × 2017 |  | 0.028 (0.019) |
| Low WPEI × 2018 |  | 0.027 (0.019) |
| Low WPEI × 2019 |  | 0.005 (0.019) |
| Middle WPEI × 2002 |  | 0.001 (0.018) |
| Middle WPEI × 2003 |  | 0.026 (0.018) |
| Middle WPEI × 2004 |  | -0.004 (0.019) |
| Middle WPEI × 2005 |  | 0.014 (0.019) |
| Middle WPEI × 2006 |  | 0.001 (0.018) |
| Middle WPEI × 2007 |  | 0.005 (0.018) |
| Middle WPEI × 2008 |  | 0.013 (0.018) |
| Middle WPEI × 2009 |  | 0.016 (0.019) |
| Middle WPEI × 2010 |  | 0.076*** (0.019) |
| Middle WPEI × 2011 |  | 0.081*** (0.019) |
| Middle WPEI × 2012 |  | 0.095*** (0.019) |
| Middle WPEI × 2013 |  | 0.076*** (0.019) |
| Middle WPEI × 2014 |  | 0.069*** (0.020) |
| Middle WPEI × 2015 |  | 0.053** (0.019) |
| Middle WPEI × 2016 |  | 0.056** (0.019) |
| Middle WPEI × 2017 |  | 0.061** (0.019) |
| Middle WPEI × 2018 |  | 0.066** (0.019) |
| Middle WPEI × 2019 |  | 0.034^†^ (0.020) |
| High WPEI × 2002 |  | 0.008 (0.019) |
| High WPEI × 2003 |  | 0.004 (0.019) |
| High WPEI × 2004 |  | 0.011 (0.019) |
| High WPEI × 2005 |  | 0.007 (0.019) |
| High WPEI × 2006 |  | -0.013 (0.019) |
| High WPEI × 2007 |  | 0.018 (0.020) |
| High WPEI × 2008 |  | -0.005 (0.020) |
| High WPEI × 2009 |  | 0.035^†^ (0.020) |
| High WPEI × 2010 |  | 0.054** (0.019) |
| High WPEI × 2011 |  | 0.052** (0.019) |
| High WPEI × 2012 |  | 0.047* (0.019) |
| High WPEI × 2013 |  | 0.050 (0.019) |
| High WPEI × 2014 |  | 0.051** (0.019) |
| High WPEI × 2015 |  | 0.043* (0.019) |
| High WPEI × 2016 |  | 0.042 (0.020) |
| High WPEI × 2017 |  | 0.070*** (0.020) |
| High WPEI × 2018 |  | 0.060** (0.020) |
| High WPEI × 2019 |  | 0.049* (0.020) |
| Very high WPEI × 2002 |  | 0.000 (0.019) |
| Very high WPEI × 2003 |  | 0.014 (0.019) |
| Very high WPEI × 2004 |  | 0.026 (0.019) |
| Very high WPEI × 2005 |  | 0.025 (0.019) |
| Very high WPEI × 2006 |  | 0.022 (0.019) |
| Very high WPEI × 2007 |  | 0.017 (0.019) |
| Very high WPEI × 2008 |  | 0.003 (0.019) |
| Very high WPEI × 2009 |  | -0.011 (0.019) |
| Very high WPEI × 2010 |  | 0.031 (0.019) |
| Very high WPEI × 2011 |  | 0.025 (0.019) |
| Very high WPEI × 2012 |  | 0.052** (0.019) |
| Very high WPEI × 2013 |  | 0.029 (0.019) |
| Very high WPEI × 2014 |  | 0.053 (0.019) |
| Very high WPEI × 2015 |  | 0.021 (0.019) |
| Very high WPEI × 2016 |  | 0.041* (0.019) |
| Very high WPEI × 2017 |  | 0.057** (0.019) |
| Very high WPEI × 2018 |  | 0.070*** (0.019) |
| Very high WPEI × 2019 |  | 0.053** (0.020) |
| Constant | 0.274*** (0.033) | 0.289*** (0.034) |
| Number of observations | 65,880 | 65,880 |
| R² | 0.367 | 0.369 |
| Adj. R² | 0.365 | 0.367 |

**Notes:** Dependent variable is PARITY; Method is OLS with country dummies to account for the fixed effects-character of the data. Coefficients for country dummies are not reported. a. Displayed are regression coefficients (standard errors in bracket). b. Reference category is ‘Very low WPEI’. c. Reference category is ‘2001’. d. Reference category is ‘Christianity’. e. Reference category is ‘Europe’. f. Reference category is ‘Very small population’. g. Reference category is ‘Very low income’. h. Reference category is ‘Walk’. i. Reference category is ‘Very low WPEI × YEAR’.
*** p<0.001, ** p<0.01, * p<0.05, ^†^p<0.1.

## Table A2. Ordered logistic regression models for Athletes

| **Independent variables** | **Model 2a^a^** | **Model 2b^a^** |
| --- | --- | --- |
| WPEI^b^ |  |  |
| Low WPEI | 1.142 (0.115) | 1.967** (0.463) |
| Middle WPEI | 0.855 (0.098) | 1.534^†^ (0.382) |
| High WPEI | 0.777* (0.098) | 1.197 (0.304) |
| Very high WPEI | 0.998 (0.144) | 0.991 (0.252) |
| YEAR^c^ |  |  |
| 2002 | 2.854 (2.967) | 5.011 (5.436) |
| 2003 | 8.117 (16.844) | 19.815 (41.988) |
| 2004 | 20.178 (62.789) | 85.520 (270.645) |
| 2005 | 44.663 (185.292) | 294.876 (1242.444) |
| 2006 | 91.941 (476.980) | 836.368 (4402.206) |
| 2007 | 254.938 (1586.298) | 2903.054 (18329.790) |
| 2008 | 551.246 (4001.603) | 9624.344 (70878.970) |
| 2009 | 3966.807 (3.291×10^4)^ | 6.430×10^4^ (5.411×10^5^) |
| 2010 | 18874.960 (1.762×10^5^) | 2.596×10^5^ (2.457×10^6^) |
| 2011 | 45952.360 (4.765×10^5^) | 1.016×10^6^ (1.070×10^7^) |
| 2012 | 1.079×10^5^ (1.231×10^6^) | 2.921×10^6^ (3.380×10^7^) |
| 2013 | 2.680×10^5^ (3.336×10^6^) | 9.995×10^6^ (1.260×10^8^) |
| 2014 | 6.818×10^5^ (9.192×10^6^) | 3.490×10^7^ (4.780×10^8^) |
| 2015 | 1.730×10^6^ (2.510×10^7^) | 1.540×10^7^ (2.270×10^9^) |
| 2016 | 4.127×10^6^ (6.420×10^7^) | 5.000×10^8^ (7.890×10^9^) |
| 2017 | 1.060×10^7^ (1.750×10^8^) | 1.410×10^9^ (2.370×10^10^) |
| 2018 | 2.330×10^7^ (4.110×10^8^) | 3.680×10^9^ (6.580×10^10^) |
| 2019 | 4.370×10^7^ (8.160×10^8^) | 1.370×10^10^ (2.590×10^11^) |
| RELIGION^d^ |  |  |
| Buddhism | 2.385 (2.502) | 2.538 (2.698) |
| Hinduism | 0.000 (0.000) | 0.000 (0.000) |
| Islam | 0.000 (0.036) | 0.007 (10.068) |
| No religion | 0.000 (7.36×10^-4^) | 2.100×10^-5^ (1.324×10^-4^) |
| Other | 1.835E+05 (2.855×10^6^) | 2.440×10^7^ (3.860×10^8^) |
| POPULATION^e^ |  |  |
| Small population | 1.493^†^ (0.337) | 2.099** (0.486) |
| Low middle population | 3.084*** (0.895) | 3.555*** (1.057) |
| Middle population | 0.979 (0.312) | 1.218 (0.397) |
| Big population | 0.662 (0.244) | 1.053 (0.398) |
| GDP PER CAPITA^f^ |  |  |
| Middle income | 0.619*** (0.039) | 0.899 (0.060) |
| Upper middle income | 0.533*** (0.046) | 1.031 (0.0961) |
| High income | 0.748* (0.085) | 1.251 (0.147) |
| NOCAGE | 0.430 (0.445) | 0.311 (0.327) |
| ASSOCIATION^g^ |  |  |
| Africa | 1.140.164 (15371.410) | 1.077×10^5^ (1.473×10^6^) |
| Asia | 1.340×10^15^ (5.830×10^16^) | 1.000×10^21^ (4.430×10^22^) |
| ConSudAtle | 7.250×10^20^ (4.360×10^22^) | 7.080×10^28^ (4.320×10^30^) |
| NACAC | 4.630×10^37^ (4.750×10^39^) | 2.380×10^51^ (2.480×10^53^) |
| Oceania | 0.127 (0.796) | 1.491 (9.479) |
| DISCIPLINE GROUP^h^ |  |  |
| Sprint | 14.629*** (0.910) | 1.482*** (0.926) |
| Middle distance running | 8.375*** (0.540) | 8.405*** (0.545) |
| Long distance running | 3.078*** (0.192) | 3.059*** (0.191) |
| Hurdles and Steeple chase | 4.958*** (0.307) | 4.948*** (0.307) |
| Jumping | 4.898*** (0.296) | 4.885*** (0.296) |
| Throwing | 4.473*** (0.270) | 4.454*** (0.270) |
| INTERACTIONS^i^ |  |  |
| Low WPEI × 2002 |  | 0.820 (0.249) |
| Low WPEI × 2003 |  | 0.832 (0.248) |
| Low WPEI × 2004 |  | 0.600^†^ (0.177) |
| Low WPEI × 2005 |  | 0.499* (0.147) |
| Low WPEI × 2006 |  | 0.462* (0.139) |
| Low WPEI × 2007 |  | 0.515* (0.158) |
| Low WPEI × 2008 |  | 0.446** (0.136) |
| Low WPEI × 2009 |  | 0.500* (0.144) |
| Low WPEI × 2010 |  | 0.679 (0.193) |
| Low WPEI × 2011 |  | 0.524* (0.149) |
| Low WPEI × 2012 |  | 0.516* (0.149) |
| Low WPEI × 2013 |  | 0.635 (0.180) |
| Low WPEI × 2014 |  | 0.605^†^ (0.170) |
| Low WPEI × 2015 |  | 0.449** (0.128) |
| Low WPEI × 2016 |  | 0.407** (0.116) |
| Low WPEI × 2017 |  | 0.612^†^ (0.176) |
| Low WPEI × 2018 |  | 0.697 (0.204) |
| Low WPEI × 2019 |  | 0.506* (0.147) |
| Middle WPEI × 2002 |  | 0.716 (0.225) |
| Middle WPEI × 2003 |  | 0.830 (0.255) |
| Middle WPEI × 2004 |  | 0.570^†^ (0.175) |
| Middle WPEI × 2005 |  | 0.613 (0.188) |
| Middle WPEI × 2006 |  | 0.582^†^ (0.178) |
| Middle WPEI × 2007 |  | 0.652 (0.200) |
| Middle WPEI × 2008 |  | 0.611 (0.187) |
| Middle WPEI × 2009 |  | 0.594^†^ (0.171) |
| Middle WPEI × 2010 |  | 0.923 (0.264) |
| Middle WPEI × 2011 |  | 0.793 (0.225) |
| Middle WPEI × 2012 |  | 0.888 (0.253) |
| Middle WPEI × 2013 |  | 0.860 (0.244) |
| Middle WPEI × 2014 |  | 0.721^†^ (0.207) |
| Middle WPEI × 2015 |  | 0.583^†^ (0.163) |
| Middle WPEI × 2016 |  | 0.601 (0.167) |
| Middle WPEI × 2017 |  | 0.680 (0.190) |
| Middle WPEI × 2018 |  | 0.861 (0.246) |
| Middle WPEI × 2019 |  | 0.565* (0.164) |
| High WPEI × 2002 |  | 0.946 (0.295) |
| High WPEI × 2003 |  | 0.808 (0.249) |
| High WPEI × 2004 |  | 0.669 (0.200) |
| High WPEI × 2005 |  | 0.537* (0.161) |
| High WPEI × 2006 |  | 0.493* (0.150) |
| High WPEI × 2007 |  | 0.626 (0.191) |
| High WPEI × 2008 |  | 0.471* (0.144) |
| High WPEI × 2009 |  | 0.922 (0.265) |
| High WPEI × 2010 |  | 1.215 (0.343) |
| High WPEI × 2011 |  | 1.084 (0.305) |
| High WPEI × 2012 |  | 1.117 (0.316) |
| High WPEI × 2013 |  | 1.166 (0.325) |
| High WPEI × 2014 |  | 1.072 (0.298) |
| High WPEI × 2015 |  | 0.946 (0.263) |
| High WPEI × 2016 |  | 0.827 (0.229) |
| High WPEI × 2017 |  | 1.165 (0.325) |
| High WPEI × 2018 |  | 1.166 (0.333) |
| High WPEI × 2019 |  | 0.915 (0.263) |
| Very high WPEI × 2002 |  | 0.676 (0.201) |
| Very high WPEI × 2003 |  | 0.626 (0.184) |
| Very high WPEI × 2004 |  | 0.484* (0.140) |
| Very high WPEI × 2005 |  | 0.422** (0.121) |
| Very high WPEI × 2006 |  | 0.471** (0.135) |
| Very high WPEI × 2007 |  | 0.434** (0.127) |
| Very high WPEI × 2008 |  | 0.401** (0.117) |
| Very high WPEI × 2009 |  | 0.838 (0.231) |
| Very high WPEI × 2010 |  | 2.177** (0.593) |
| Very high WPEI × 2011 |  | 1.720* (0.468) |
| Very high WPEI × 2012 |  | 2.388** (0.654) |
| Very high WPEI × 2013 |  | 2.053** (0.554) |
| Very high WPEI × 2014 |  | 2.635*** (0.713) |
| Very high WPEI × 2015 |  | 1.758* (0.474) |
| Very high WPEI × 2016 |  | 2.110** (0.564) |
| Very high WPEI × 2017 |  | 2.647*** (0.713) |
| Very high WPEI × 2018 |  | 3.360*** (0.926) |
| Very high WPEI × 2019 |  | 1.861* (0.516) |
| Cut1 | -5.281 (8.304) | -7.078 (8.421) |
| Cut2 | -1.276 (8.304) | -2.958 (8.420) |
| Number of observations | 63,807 | 63,807 |
| AIC | 63132.62 | 62594.93 |
| Pseudo R² | 0.455 | 0.461 |

**Notes:** Dependent variable is ATHLETES; Method is ordered logistic regression with country dummies to account for the fixed effects-character of the data. Coefficients for country dummies are not reported. a. Displayed are odds ratios (standard errors in bracket). b. Reference category is ‘Very low WPEI’. c. Reference category is ‘2001’. d. Reference category is ‘Christianity’. e. Reference category is ‘Europe’. f. Reference category is ‘Very small population’. g. Reference category is ‘Very low income’. h. Reference category is ‘Walk’. i. Reference category is ‘Very low WPEI × YEAR’.
*** p<0.001, ** p<0.01, * p<0.05, ^†^p<0.1.

## Table A3. Influence of the interaction between ATHLETICS and YEAR on PARITY

| **Independent variables** | **Parity (FE)^a^** |
| --- | --- |
| ATHLETES^b^ |  |
| < 0.1 women’s athletes | 0.639*** (0.005) |
| ≥ 0.1 women’s athletes | 0.713*** (0.015) |
| YEAR^c^ |  |
| 2002 | -0.001 (0.004) |
| 2003 | -0.003 (0.004) |
| 2004 | -0.002 (0.004) |
| 2005 | -0.003 (0.004) |
| 2006 | -0.003 (0.004) |
| 2007 | -0.003 (0.004) |
| 2008 | -0.003 (0.004) |
| 2009 | -0.007 (0.004) |
| 2010 | -0.009 (0.004) |
| 2011 | -0.009 (0.004) |
| 2012 | -0.009 (0.004) |
| 2013 | -0.008 (0.004) |
| 2014 | -0.009 (0.004) |
| 2015 | -0.009 (0.004) |
| 2016 | -0.009 (0.004) |
| 2017 | -0.009 (0.004) |
| 2018 | -0.010 (0.004) |
| 2019 | -0.010 (0.004) |
| INTERACTIONS |  |
| < 0.1 women’s athletes × 2002 | 0.002 (0.007) |
| < 0.1 women’s athletes × 2003 | 0.010 (0.007) |
| < 0.1 women’s athletes × 2004 | 0.014 (0.007) |
| < 0.1 women’s athletes × 2005 | 0.012 (0.007) |
| < 0.1 women’s athletes × 2006 | 0.004 (0.007) |
| < 0.1 women’s athletes × 2007 | 0.011 (0.007) |
| < 0.1 women’s athletes × 2008 | 0.017 (0.007) |
| < 0.1 women’s athletes × 2009 | -0.078*** (0.007) |
| < 0.1 women’s athletes × 200.1 | -0.068*** (0.007) |
| < 0.1 women’s athletes × 2011 | -0.070*** (0.007) |
| < 0.1 women’s athletes × 2012 | -0.070*** (0.007) |
| < 0.1 women’s athletes × 2013 | -0.062*** (0.007) |
| < 0.1 women’s athletes × 2014 | -0.062*** (0.007) |
| < 0.1 women’s athletes × 2015 | -0.055*** (0.007) |
| < 0.1 women’s athletes × 2016 | -0.064*** (0.007) |
| < 0.1 women’s athletes × 2017 | -0.066*** (0.007) |
| < 0.1 women’s athletes × 2018 | -0.069*** (0.007) |
| < 0.1 women’s athletes × 2019 | -0.058*** (0.007) |
|  |  |
| ≥ 0.1 women’s athletes × 2002 | -0.003 (0.020) |
| ≥ 0.1 women’s athletes × 2003 | -0.027 (0.020) |
| ≥ 0.1 women’s athletes × 2004 | 0.012 (0.019) |
| ≥ 0.1 women’s athletes × 2005 | 0.009 (0.020) |
| ≥ 0.1 women’s athletes × 2006 | 0.009 (0.020) |
| ≥ 0.1 women’s athletes × 2007 | 0.033 (0.020) |
| ≥ 0.1 women’s athletes × 2008 | 0.012 (0.020) |
| ≥ 0.1 women’s athletes × 2009 | -0.117*** (0.017) |
| ≥ 0.1 women’s athletes × 200.1 | -0.105*** (0.016) |
| ≥ 0.1 women’s athletes × 2011 | -0.110*** (0.016) |
| ≥ 0.1 women’s athletes × 2012 | -0.106*** (0.016) |
| ≥ 0.1 women’s athletes × 2013 | -0.113*** (0.016) |
| ≥ 0.1 women’s athletes × 2014 | -0.120*** (0.016) |
| ≥ 0.1 women’s athletes × 2015 | -0.115*** (0.016) |
| ≥ 0.1 women’s athletes × 2016 | -0.106*** (0.016) |
| ≥ 0.1 women’s athletes × 2017 | -0.100*** (0.016) |
| ≥ 0.1 women’s athletes × 2018 | -0.094*** (0.016) |
| ≥ 0.1 women’s athletes × 2019 | -0.088*** (0.016)) |
| Constant | 0.006 (0.007) |
| Number of observations | 74,847 |
| R² | 0.794 |
| Adj R² | 0.793 |

**Notes:** Dependent variable is PARITY; Method is OLS with country dummies to account for the fixed effects-character of the data. Coefficients for country dummies are not reported. a. Displayed are regression coefficients (standard errors in bracket). b. Reference category is ‘No women’s athletes’. c. Reference category is ‘2001’. d. Reference category is ‘No women’s athletes × YEAR’.
*** p<0.001, ** p<0.01, * p<0.05, ^†^p<0.1.

## Table A4. Ordered logistic regression models for Hosting

| **Independent variables** | **Model 3a^a^** | **Model 3b^a^** |
| --- | --- | --- |
| WPEI^b^ |  |  |
| Low WPEI | 1.470** (0.190) | 4.380*** (1.747) |
| Middle WPEI | 1.443* (0.208) | 3.359** (1.375) |
| High WPEI | 1.199 (0.188) | 3.224** (1.329) |
| Very high WPEI | 1.953*** (0.338) | 4.039** (1.656) |
| YEAR^c^ |  |  |
| 2002 | 1.401 (0.943) | 2.716 (2.268) |
| 2003 | 1.550 (2.076) | 5.397 (7.732) |
| 2004 | 1.564 (3.139) | 12.463 (25.948) |
| 2005 | 1.550 (4.147) | 7.489 (20.644) |
| 2006 | 1.374 (4.602) | 20.976 (71.759) |
| 2007 | 1.434 (5.751) | 24.712 (101.215) |
| 2008 | 1.096 (5.128) | 23.435 (111.841) |
| 2009 | 3.615 (19.335) | 134.580 (732.908) |
| 2010 | 6.191 (37.252) | 181.033 (1108.468) |
| 2011 | 5.871 (39.249) | 275.233 (1871.690) |
| 2012 | 6.509 (47.869) | 267.045 (1997.054) |
| 2013 | 5.720 (45.886) | 259.624 (2117.575) |
| 2014 | 5.950 (51.710) | 237.110 (2094.792) |
| 2015 | 6.893 (64.514) | 934.490 (8889.398) |
| 2016 | 6.403 (64.207) | 512.338 (5221.275) |
| 2017 | 8.080 (86.424) | 648.945 (7053.800) |
| 2018 | 7.717 (87.705) | 743.207 (8582.922) |
| 2019 | 11.056 (133.042) | 2877.926 (35187.250) |
| RELIGION^d^ |  |  |
| Buddhism | 1.373 (0.944) | 1.704 (1.189) |
| Hinduism | 0.015 (0.121) | 0.001 (0.007) |
| Islam | 0.000 (0.000) | 0.000 (0.000) |
| No religion | 0.056 (0.227) | 0.015 (0.061) |
| Other | 0.000 (0.000) | 0.000 (0.002) |
| POPULATION^e^ |  |  |
| Small population | 0.824 (0.294) | 0.810 (0.292) |
| Low middle population | 0.925 (0.365) | 0.928 (0.372) |
| Middle population | 0.524 (0.222) | 0.617 (0.265) |
| Big population | 0.370* (0.171) | 0.530 (0.250) |
| GDP PER CAPITA^f^ |  |  |
| Middle income | 0.895 (0.069) | 1.039 (0.086) |
| Upper middle income | 0.985 (0.100) | 1.291* (0.145) |
| High income | 1.552** (0.202) | 2.038*** (0.278) |
| NOCAGE | 1.007 (0.673) | 0.776 (0.527) |
| ASSOCIATION^g^ |  |  |
| Africa | 0.003 (0.022) | 0.077 (0.679) |
| Asia | 0.145 (4.071) | 7.18×10^3^ (2.05×10^5^) |
| ConSudAtle | 0.132 (5.102) | 4.01×10^5^ (1.58×10^7^) |
| NACAC | 0.930 (61.570) | 9.19×10^10^ (6.18×10^12^) |
| Oceania | 0.002 (0.010) | 0.013 (0.052) |
| DISCIPLINE GROUP^h^ |  |  |
| Sprint | 10.306*** (0.702) | 10.428 (0.713) |
| Middle distance running | 5.677*** (0.404) | 5.714 (0.408) |
| Long distance running | 2.390*** (0.164) | 2.397 (0.165) |
| Hurdles and Steeple chase | 4.174*** (0.284) | 4.199 (0.287) |
| Jumping | 5.324*** (0.353) | 5.362 (0.357) |
| Throwing | 5.570*** (0.369) | 5.608 (0.374) |
| INTERACTIONS^i^ |  |  |
| Low WPEI × 2002 |  | 0.731 (0.376) |
| Low WPEI × 2003 |  | 0.390^†^ (0.190) |
| Low WPEI × 2004 |  | 0.227** (0.106) |
| Low WPEI × 2005 |  | 0.547 (0.275) |
| Low WPEI × 2006 |  | 0.175*** (0.081) |
| Low WPEI × 2007 |  | 0.315* (0.147) |
| Low WPEI × 2008 |  | 0.284** (0.136) |
| Low WPEI × 2009 |  | 0.126*** (0.056) |
| Low WPEI × 2010 |  | 0.313** (0.138) |
| Low WPEI × 2011 |  | 0.172*** (0.076) |
| Low WPEI × 2012 |  | 0.245** (0.109) |
| Low WPEI × 2013 |  | 0.345* (0.154) |
| Low WPEI × 2014 |  | 0.490 (0.220) |
| Low WPEI × 2015 |  | 0.167*** (0.074) |
| Low WPEI × 2016 |  | 0.224** (0.102) |
| Low WPEI × 2017 |  | 0.814 (0.367) |
| Low WPEI × 2018 |  | 0.556 (0.256) |
| Low WPEI × 2019 |  | 0.201*** (0.090) |
| Middle WPEI × 2002 |  | 0.541 (0.282) |
| Middle WPEI × 2003 |  | 0.446 (0.219) |
| Middle WPEI × 2004 |  | 0.224 (0.106) |
| Middle WPEI × 2005 |  | 0.559 (0.286) |
| Middle WPEI × 2006 |  | 0.284** (0.132) |
| Middle WPEI × 2007 |  | 0.290** (0.136) |
| Middle WPEI × 2008 |  | 0.308* (0.148) |
| Middle WPEI × 2009 |  | 0.172*** (0.077) |
| Middle WPEI × 2010 |  | 0.359* (0.159) |
| Middle WPEI × 2011 |  | 0.273** (0.121) |
| Middle WPEI × 2012 |  | 0.437^†^ (0.194) |
| Middle WPEI × 2013 |  | 0.424^†^ (0.190) |
| Middle WPEI × 2014 |  | 0.789 (0.360) |
| Middle WPEI × 2015 |  | 0.215** (0.095) |
| Middle WPEI × 2016 |  | 0.764 (0.340) |
| Middle WPEI × 2017 |  | 0.765 (0.342) |
| Middle WPEI × 2018 |  | 1.019 (0.466) |
| Middle WPEI × 2019 |  | 0.384 (0.172) |
| High WPEI × 2002 |  | 0.738 (0.382) |
| High WPEI × 2003 |  | 0.635 (0.310) |
| High WPEI × 2004 |  | 0.298** (0.138) |
| High WPEI × 2005 |  | 0.513 (0.259) |
| High WPEI × 2006 |  | 0.213** (0.099) |
| High WPEI × 2007 |  | 0.242** (0.113) |
| High WPEI × 2008 |  | 0.218** (0.105) |
| High WPEI × 2009 |  | 0.255** (0.112) |
| High WPEI × 2010 |  | 0.269** (0.118) |
| High WPEI × 2011 |  | 0.219** (0.096) |
| High WPEI × 2012 |  | 0.404* (0.178) |
| High WPEI × 2013 |  | 0.381* (0.168) |
| High WPEI × 2014 |  | 0.500 (0.222) |
| High WPEI × 2015 |  | 0.192*** (0.084) |
| High WPEI × 2016 |  | 0.492 (0.217) |
| High WPEI × 2017 |  | 0.681 (0.303) |
| High WPEI × 2018 |  | 0.635 (0.289) |
| High WPEI × 2019 |  | 0.334 (0.148) |
| Very high WPEI × 2002 |  | 0.641 (0.324) |
| Very high WPEI × 2003 |  | 0.454^†^ (0.216) |
| Very high WPEI × 2004 |  | 0.258** (0.117) |
| Very high WPEI × 2005 |  | 0.612 (0.301) |
| Very high WPEI × 2006 |  | 0.227** (0.101) |
| Very high WPEI × 2007 |  | 0.222** (0.100) |
| Very high WPEI × 2008 |  | 0.266** (0.123) |
| Very high WPEI × 2009 |  | 0.204*** (0.087) |
| Very high WPEI × 2010 |  | 0.371* (0.159) |
| Very high WPEI × 2011 |  | 0.349* (0.149) |
| Very high WPEI × 2012 |  | 0.465^†^ (0.200) |
| Very high WPEI × 2013 |  | 0.691 (0.297) |
| Very high WPEI × 2014 |  | 1.156 (0.503) |
| Very high WPEI × 2015 |  | 0.421 (0.179) |
| Very high WPEI × 2016 |  | 0.878 (0.377) |
| Very high WPEI × 2017 |  | 0.919 (0.398) |
| Very high WPEI × 2018 |  | 1.141 (0.505) |
| Very high WPEI × 2019 |  | 0.611 (0.264) |
| Cut1 | 0.915 (0.365) | -0.258 (5.459) |
| Cut2 | 5.344 (5.365) | 4.237 (5.459) |
| Number of observations | 63,839 | 63,839 |
| AIC | 50743.24 | 50540.58 |
| Pseudo R² | 0.493 | 0.497 |

**Notes:** Dependent variable is HOSTINGS; Method is ordered logistic regression with country dummies to account for the fixed effects-character of the data. Coefficients for country dummies are not reported. a. Displayed are odds ratios (standard errors in bracket). b. Reference category is ‘Very low WPEI’. c. Reference category is ‘2001’. d. Reference category is ‘Christianity’. e. Reference category is ‘Europe’. f. Reference category is ‘Very small population’. g. Reference category is ‘Very low income’. h. Reference category is ‘Walk’. i. Reference category is ‘Very low WPEI × YEAR’.
*** p<0.001, ** p<0.01, * p<0.05, ^†^p<0.1.

## Table A5. Tobit regression models for Disciplines

|  | **Model 4a (FE)^a^** | **Model 4b (FE)^a^** |
| --- | --- | --- |
| WPEI^b^ |  |  |
| Low WPEI | 2.583*** (0.375) | -0.281 (0.788) |
| Middle WPEI | 4.156*** (0.375) | -1.322 (0.873) |
| High WPEI | 6.410*** (0.407) | -1.390 (0.885) |
| Very high WPEI | 8.836*** (0.513) | -0.414 (0.966) |
| YEAR^c^ |  |  |
| 2002 | 0.605 (0.674) | 1.165 (2.655) |
| 2003 | 1.140 (0.670) | 1.885 (5.133) |
| 2004 | 0.913 (0.668) | 2.584 (7.651) |
| 2005 | 0.251 (0.673) | 2.466 (10.181) |
| 2006 | -0.579 (0.675) | 2.473 (12.711) |
| 2007 | -0.327 (0.671) | 3.092 (15.243) |
| 2008 | -0.765 (0.670) | 3.089 (17.777) |
| 2009 | 2.492*** (0.665) | 5.091 (20.312) |
| 2010 | 3.998*** (0.665) | 5.975 (22.847) |
| 2011 | 3.957*** (0.667) | 6.610 (25.382) |
| 2012 | 3.536*** (0.665) | 6.556 (27.918) |
| 2013 | 3.604*** (0.667) | 6.719 (30.454) |
| 2014 | 3.568*** (0.669) | 6.932 (32.991) |
| 2015 | 3.913*** (0.670) | 8.130 (35.528) |
| 2016 | 3.783*** (0.665) | 8.710 (38.065) |
| 2017 | 4.191*** (0.667) | 8.234 (40.602) |
| 2018 | 3.703*** (0.667) | 7.825 (43.139) |
| 2019 | 2.977*** (0.667) | 8.352 (45.676) |
| RELIGION^d^ |  |  |
| Buddhism | -2.044** (0.654) | -1.612 (2.661) |
| Hinduism | -0.628 (0.845) | -17.224 (30.459) |
| Islam | -1.804*** (0.322) | -29.695 (1634.722) |
| No religion | 0.057 (0.638) | -11.893 (15.244) |
| Other | 1.256 (0.568) | 0.191 (38.065) |
| POPULATION^e^ |  |  |
| Small population | 4.427*** (0.417) | 2.612** (0.908) |
| Low middle population | 9.263*** (0.395) | 1.263 (1.117) |
| Middle population | 13.333*** (0.600) | -0.632 (1.291) |
| Big population | 16.607*** (0.639) | 0.001 (1.597) |
| GDP PER CAPITA^f^ |  |  |
| Middle income | -1.138** (0.380) | -1.248*** (0.335) |
| Upper middle income | -3.716*** (0.412) | -2.500*** (0.423) |
| High income | -6.129*** (0.454) | -2.549*** (0.527) |
| NOCAGE | 0.050 (0.005) | -0.109 (2.537) |
| ASSOCIATION^g^ |  |  |
| Africa | -4.280*** (0.412) | -31.058 (1574.711) |
| Asia | -4.987*** (0.435) | -18.174 (1577.968) |
| ConSudAtle | -3.213*** (0.494) | -16.956 (1581.228) |
| NACAC | -3.797*** (0.450) | 9.030 (2221.854) |
| Oceania | -1.146 (0.772) | -40.276 (1574.440) |
| INTERACTIONS^h^ |  |  |
| Low WPEI × 2002 |  | 0.077 (0.991) |
| Low WPEI × 2003 |  | 0.132 (0.985) |
| Low WPEI × 2004 |  | -0.589 (0.993) |
| Low WPEI × 2005 |  | -1.031 (1.015) |
| Low WPEI × 2006 |  | -1.737^†^ (1.035) |
| Low WPEI × 2007 |  | -1.410 (1.047) |
| Low WPEI × 2008 |  | -1.742^†^ (1.051) |
| Low WPEI × 2009 |  | -0.374 (1.012) |
| Low WPEI × 2010 |  | 0.850 (1.003) |
| Low WPEI × 2011 |  | -0.112 (1.013) |
| Low WPEI × 2012 |  | -0.421 (1.016) |
| Low WPEI × 2013 |  | 0.765 (1.006) |
| Low WPEI × 2014 |  | 0.464 (1.010) |
| Low WPEI × 2015 |  | -0.495 (1.035) |
| Low WPEI × 2016 |  | -1.464 (1.041) |
| Low WPEI × 2017 |  | 0.309 (1.029) |
| Low WPEI × 2018 |  | 0.643 (1.036) |
| Low WPEI × 2019 |  | -0.450 (1.043) |
| Middle WPEI × 2002 |  | -0.414 (1.063) |
| Middle WPEI × 2003 |  | 0.412 (1.045) |
| Middle WPEI × 2004 |  | -0.407 (1.055) |
| Middle WPEI × 2005 |  | 0.326 (1.081) |
| Middle WPEI × 2006 |  | 0.035 (1.058) |
| Middle WPEI × 2007 |  | -0.106 (1.049) |
| Middle WPEI × 2008 |  | -0.030 (1.047) |
| Middle WPEI × 2009 |  | 0.443 (1.023) |
| Middle WPEI × 2010 |  | 2.180* (1.026) |
| Middle WPEI × 2011 |  | 1.729^†^ (1.032) |
| Middle WPEI × 2012 |  | 1.861^†^ (1.041) |
| Middle WPEI × 2013 |  | 1.787^†^ (1.033) |
| Middle WPEI × 2014 |  | 1.577 (1.058) |
| Middle WPEI × 2015 |  | 0.909 (1.030) |
| Middle WPEI × 2016 |  | 0.552 (1.023) |
| Middle WPEI × 2017 |  | 1.192 (1.022) |
| Middle WPEI × 2018 |  | 1.750^†^ (1.043) |
| Middle WPEI × 2019 |  | 0.154 (1.078) |
| High WPEI × 2002 |  | -0.161 (1.034) |
| High WPEI × 2003 |  | -0.533 (1.027) |
| High WPEI × 2004 |  | -0.678 (1.016) |
| High WPEI × 2005 |  | -0.945 (1.045) |
| High WPEI × 2006 |  | -1.783^†^ (1.049) |
| High WPEI × 2007 |  | -1.374 (1.048) |
| High WPEI × 2008 |  | -1.600 (1.046) |
| High WPEI × 2009 |  | 1.666 (1.050) |
| High WPEI × 2010 |  | 2.258* (1.021) |
| High WPEI × 2011 |  | 2.106 (1.017) |
| High WPEI × 2012 |  | 1.667^†^ (1.003) |
| High WPEI × 2013 |  | 1.800^†^ (1.001) |
| High WPEI × 2014 |  | 1.676 (1.007) |
| High WPEI × 2015 |  | 1.174 (1.016) |
| High WPEI × 2016 |  | 0.394 (1.009) |
| High WPEI × 2017 |  | 2.038* (1.033) |
| High WPEI × 2018 |  | 1.591 (1.047) |
| High WPEI × 2019 |  | 0.253 (1.067) |
| Very high WPEI × 2002 |  | -0.835 (1.065) |
| Very high WPEI × 2003 |  | -0.576 (1.065) |
| Very high WPEI × 2004 |  | -0.885 (1.069) |
| Very high WPEI × 2005 |  | -1.289 (1.080) |
| Very high WPEI × 2006 |  | -1.440 (1.066) |
| Very high WPEI × 2007 |  | -2.014^†^ (1.058) |
| Very high WPEI × 2008 |  | -2.490* (1.062) |
| Very high WPEI × 2009 |  | -0.229 (1.055) |
| Very high WPEI × 2010 |  | 0.952 (1.061) |
| Very high WPEI × 2011 |  | 0.573 (1.077) |
| Very high WPEI × 2012 |  | 0.861 (1.063) |
| Very high WPEI × 2013 |  | 0.848 (1.058) |
| Very high WPEI × 2014 |  | 1.596 (1.075) |
| Very high WPEI × 2015 |  | -0.115 (1.078) |
| Very high WPEI × 2016 |  | -0.822 (1.042) |
| Very high WPEI × 2017 |  | 0.570 (1.058) |
| Very high WPEI × 2018 |  | 0.685 (1.063) |
| Very high WPEI × 2019 |  | -0.107 (1.095) |
| Constant | -2.771** (0.800) | 36.237 (1574.497) |
| Var(e.DISCIPLINES) | 31.310 (1.006) | 5.754 (0.178) |
| Multiple R² | 0.685 | 0.819 |
| LR chi² | 3767.36 | 8175.41 |
| Pseudo R² | 0.202 | 0.438 |
| Prob>chi² | 0.000 | 0.000 |
| Left-censored obs. | 690 | 690 |
| Right-censored obs. | 468 | 468 |
| Observations | 3,290 | 3,290 |

**Notes:** Dependent variable is DISCIPLINES; Method is tobit regressions with country dummies to account for the fixed effects-character of the data due to the truncated dependent variable. Coefficients for country dummies are not reported. a. Displayed are tobit regression coefficients (standard errors in bracket). b. Reference category is ‘Very low WPEI’. c. Reference category is ‘2001’. d. Reference category is ‘Christianity’. e. Reference category is ‘Very small population’. f. Reference category is ‘Very low income’. g. Reference category is ‘Europe’. h. ‘Very low WPEI × YEAR’. *** p<0.001, ** p<0.01, * p<0.05, ^†^p<0.1.

## Table A6. Robustness checks for Ordered logistic regressions for Athletes

|  | **WPEI = 1^a^** | **WPEI = 2^a^** | **WPEI = 3^a^** | **WPEI = 4^a^** | **WPEI = 5^a^** |
| --- | --- | --- | --- | --- | --- |
| YEAR^b^ |  |  |  |  |  |
| 2002 | 1.269 (1620.617) | 1.481* (0.237) | 2.580 (2.809) | 1.420 (0.230) | 1.114 (0.153) |
| 2003 | 1.305 (3332.371) | 2.200*** (0.360) | 9.095 (19.596) | 1.651** (0.276) | 1.263 (0.174) |
| 2004 | 1.564 (5989.304) | 2.348*** (0.409) | 18.986 (61.260) | 2.086*** (0.353) | 1.286 (0.177) |
| 2005 | 1.474 (7527.863) | 2.421*** (0.440) | 54.131 (232.729) | 2.157*** (0.404) | 1.159 (0.158) |
| 2006 | 1.169 (7464.355) | 2.245*** (0.441) | 105.741 (569.415) | 2.065*** (0.425) | 1.121 (0.150) |
| 2007 | 0.935 (7162.078) | 3.387*** (0.722) | 291.428 (1877.577) | 3.158*** (0.695) | 1.122 (0.154) |
| 2008 | 0.812 (7261.438) | 3.349*** (0.759) | 643.587 (4837.242) | 2.782*** (0.658) | 1.060 (0.144) |
| 2009 | 1.514 (15466.260) | 9.548*** (2.227) | 3170.131 (27229.480) | 11.375*** (2.828) | 4.687*** (0.641) |
| 2010 | 1.549 (17804.080) | 20.148*** (4.992) | 1.549E+04 (1.497E+05) | 20.234*** (5.426) | 16.304*** (2.260) |
| 2011 | 1.504 (19207.670) | 22.704*** (6.160) | 4.020E+04 (4.316E+05) | 24.454*** (6.997) | 14.968*** (2.090) |
| 2012 | 1.242 (17440.290) | 21.978*** (6.325) | 9.910E+04 (1.171E+06) | 25.530*** (7.793) | 18.264*** (2.592) |
| 2013 | 1.162 (17803.770) | 35.612*** (10.959) | 2.592E+05 (3.340E+06) | 32.331*** (10.591) | 16.303*** (2.274) |
| 2014 | 1.132 (18796.960) | 42.209*** (13.588) | 5.739E+05 (8.012E+06) | 36.084*** (12.407) | 23.297*** (3.374) |
| 2015 | 1.433 (25608.470) | 50.981*** (17.700) | 1.485E+06 (2.230E+07) | 45.912*** (16.605) | 20.618*** (3.020) |
| 2016 | 1.286 (24627.420) | 52.192*** (18.747) | 3.685E+06 (5.930E+07) | 46.481*** (17.843) | 23.274*** (3.418) |
| 2017 | 0.966 (19738.790) | 74.900*** (28.196) | 9.176E+06 (1.580E+08) | 61.077*** (24.648) | 25.887*** (3.880) |
| 2018 | 0.691 (15001.620) | 73.185*** (28.891) | 2.220E+07 (4.050E+08) | 57.356*** (24.258) | 27.363*** (4.123) |
| 2019 | 0.716 (16444.410) | 71.133*** (29.212) | 3.900E+07 (7.530E+08) | 56.242*** (24.804) | 17.617*** (2.672) |
| RELIGION^c^ |  |  |  |  |  |
| Buddhism | 66.634 (3062822.000) | 0.001*** (0.001) | 1.556E+00 (1.698E+00) | 0.048*** (0.036) | empty |
| Hinduism | 0.000 (0.096) | 17.480 (23.523) | 1.370E-07 (1.770E-06) | 0.787 (0.639) | empty |
| Islam | 0.000 (0.001) | 110.598*** (53.938) | 3.560E+09 (8.790E+10) | 2.739*** (0.774) | 0.006*** (0.004) |
| No religion | empty | 0.506 (0.221) | 8.720E-16 (3.930E-14) | 0.035*** (0.016) | 1.961 (1.417) |
| Other | empty | 5.732*** (2.301) | 5.838E+05 (9.408E+06) | 6.725*** (2.621) | 64.259*** (51.152) |
| POPULATION^d^ |  |  |  |  |  |
| Small population | 2.835** (0.868) | 1.270E+07 (7.310E+10) | 3.344E+05 (4.810E+08) | 82.880*** (82.209) | 2.152 (0.900) |
| Low middle population | 3.751** (1.602) | 1.510E+07 (8.740E+10) | 4.792E+04 (6.900E+07) | 313.080*** (171.771) | 6.135*** (1.709) |
| Middle population | 3.957* (2.139) | 1.000E+14 (5.810E+17) | 7.188E+03 (1.030E+07) | 139.011*** (78.983) | 1.259 (0.525) |
| Big population | 66.114 (1144727.000) | 8.560E+13 (4.950E+17) | 7.740E+34 (1.120E+38) | 114.179*** (71.087) | 1.449 (0.687) |
| GDP PER CAPITA^e^ |  |  |  |  |  |
| Middle income | 1.012 (0.146) | 1.086 (0.150) | 1.295 (0.211) | 0.541** (0.100) | 8.946*** (4.048) |
| Upper middle income | 4.508*** (1.540) | 1.313 (0.264) | 1.936** (0.430) | 0.446*** (0.103) | 2.548* (1.021) |
| High income | 5.529*** (2.637) | 0.074** (0.070) | 1.486 (0.618) | 0.490** (0.134) | 3.757** (1.459) |
| NOCAGE | 1.142 (1458.144) | 0.886*** (0.018) | 0.415 (0.445) | 0.897*** (0.020) | 1.018*** (0.004) |
| ASSOCIATION^f^ |  |  |  |  |  |
| Africa | 1.756 (24657.570) | 17.265*** (11.928) | 4.522E+02 (6.317E+03) | 3.593 (2.545) | 4.299E-01** (1.346E-01) |
| Asia | 0.005 (489.264) | 0.234 (0.244) | 3.930E+15 (1.770E+17) | 23.068*** (19.887) | 3.134E-03*** (2.314E-03) |
| ConSudAtle | empty | 53.662*** (40.214) | 8.840E+20 (5.510E+22) | 362.473*** (446.565) | 0.009*** (0.003) |
| NACAC | 0.020 (1501.878) | 41.106*** (37.333) | 3.830E+14 (1.440E+16) | 65.621*** (40.939) | 2.592* (0.966) |
| Oceania | omitted | 8.415E+05 (4.860E+09) | empty | empty | 1.356 (0.475) |
| DISCIPLINE GROUP^g^ |  |  |  |  |  |
| Sprint | 32.900*** (10.150) | 6.187*** (0.919) | 7.090*** (0.970) | 14.085*** (1.698) | 38.609*** (4.493) |
| Middle distance running | 21.505*** (6.744) | 3.618*** (0.564) | 4.097*** (0.586) | 6.539*** (0.817) | 22.479*** (2.691) |
| Long distance running | 8.943*** (2.798) | 1.659** (0.250) | 2.194*** (0.304) | 2.647*** (0.318) | 4.675*** (0.513) |
| Hurdles and Steeple chase | 9.835*** (3.065) | 1.982*** (0.297) | 2.524*** (0.348) | 4.255*** (0.508) | 12.727*** (1.420) |
| Jumping | 6.863*** (2.126) | 1.649** (0.241) | 2.485*** (0.334) | 5.167*** (0.603) | 12.653*** (1.376) |
| Throwing | 8.809*** (2.720) | 1.580** (0.231) | 2.068*** (0.279)) | 4.596*** (0.535)) | 10.797*** (1.168) |
| Cut1 | 12.442 (22982.39) | 19.796 (5780.423) | 2.435 (1439.696) | 5.665 (0.403) | 4.550 (0.603) |
| Cut2 | 15.602 (22982.39) | 25.522 (5780.423) | 7.600 (1439.696) | 9.167 (0.409) | 8.820 (0.609) |
| Number of observations | 12,760 | 12,727 | 13,020 | 12,560 | 12,740 |
| AIC | 6919.352 | 9221.049 | 11786.88 | 16566.42 | 16556.39 |
| Pseudo R² | 0.345 | 0.449 | 0.370 | 0.306 | 0.355 |

**Notes:** Dependent variable is ATHLETES; Method is ordered logistic regression with country dummies to account for the fixed effects-character of the data. Coefficients for country dummies are not reported. a. Displayed are odds ratios (standard errors in bracket). b. Reference category is ‘Very low WPEI’. c. Reference category is ‘2001’. d. Reference category is ‘Christianity’. e. Reference category is ‘Europe’. f. Reference category is ‘Very small population’. g. Reference category is ‘Very low income’. h. Reference category is ‘Walk’.
*** p<0.001, ** p<0.01, * p<0.05, ^†^p<0.1.

## Table A7. Robustness checks for Ordered logistic regressions for Hosting

|  | **WPEI = 1^a^** | **WPEI = 2 ^a^** | **WPEI = 3 ^a^** | **WPEI = 4 ^a^** | **WPEI = 5 ^a^** |
| --- | --- | --- | --- | --- | --- |
| YEAR^b^ |  |  |  |  |  |
| 2002 | 2.745 (4419.774) | 1.649** (0.313) | 0.927 (0.684) | 1.590* (0.297) | 1.358* (0.184) |
| 2003 | 3.995 (12863.940) | 1.277 (0.272) | 1.017 (1.458) | 2.274*** (0.436) | 1.467** (0.199) |
| 2004 | 6.488 (31339.780) | 1.422 (0.350) | 0.790 (1.690) | 1.989** (0.394) | 1.489** (0.203) |
| 2005 | 2.755 (17741.970) | 1.654 (0.463) | 0.806 (2.296) | 1.858** (0.408) | 1.612*** (0.216) |
| 2006 | 6.516 (52462.810) | 1.129 (0.365) | 0.690 (2.470) | 1.839* (0.447) | 1.305* (0.172) |
| 2007 | 4.701 (45420.130) | 2.006 (0.738) | 0.470 (2.003) | 2.065** (0.542) | 1.140 (0.153) |
| 2008 | 3.144 (35437.230) | 1.207 (0.501) | 0.288 (1.431) | 1.509 (0.431) | 1.010 (0.135) |
| 2009 | 13.350 (171965.000) | 2.619* (1.201) | 0.671 (3.809) | 7.914*** (2.386) | 3.218*** (0.437) |
| 2010 | 11.358 (164601.600) | 8.239*** (4.155) | 1.414 (9.032) | 9.757*** (3.193) | 5.928*** (0.809) |
| 2011 | 12.625 (203287.700) | 4.703** (2.627) | 1.050 (7.447) | 10.270*** (3.611) | 6.316*** (0.870) |
| 2012 | 9.139 (161876.700) | 5.280** (3.217) | 1.091 (8.518) | 15.491*** (5.841) | 6.112*** (0.845) |
| 2013 | 6.188 (119568.600) | 5.713** (3.771) | 0.743 (6.329) | 12.491*** (5.062) | 6.768*** (0.925) |
| 2014 | 4.153 (86942.800) | 5.929* (4.202) | 0.833 (7.691) | 12.016*** (5.128) | 8.001*** (1.123) |
| 2015 | 12.137 (273589.800) | 5.778* (4.411) | 0.509 (5.059) | 14.967*** (6.747) | 8.694*** (1.239) |
| 2016 | 5.783 (139679.300) | 2.857 (2.317) | 0.686 (7.307) | 18.106*** (8.681) | 7.369*** (1.050) |
| 2017 | 5.099 (131363.000) | 13.957** (12.010) | 0.569 (6.466) | 25.387*** (12.844) | 7.823*** (1.131) |
| 2018 | 4.251 (116365.100) | 7.142* (6.512) | 0.530 (6.398) | 22.833*** (12.140) | 8.852*** (1.286) |
| 2019 | 10.724 (310824.700) | 9.060* (8.706) | 0.551 (7.045) | 38.353*** (21.298) | 14.569*** (2.174) |
| RELIGION |  |  |  |  |  |
| Buddhism | 23.443 (1358934.000) | 0.723 (1.485) | 0.984 (0.724) | 7.83E+04 (1.33E+08) | empty |
| Hinduism | 0.000 (0.050) | 0.000 (0.000) | 0.218 (1.858) | 3.16E+00 (3.19E+00) | empty |
| Islam | 0.000 (0.002) | 244.905*** (194.257) | 0.313 (5.114) | 1.22E+00 (4.59E-01) | 7.17E-03*** (5.09E-03) |
| No religion | empty | 24.645*** (17.651) | 4.776E+04 (1.426E+06) | 2.04E+05 (3.46E+08) | 3.35E+00 (2.82E+00) |
| Other | empty | 0.000 (0.000) | 0.000 (0.000) | 1.47E+07 (2.48E+10) | 6.62E+01*** (6.02E+01) |
| POPULATION |  |  |  |  |  |
| Small population | 1.890E+07 (4.940E+10) | 1.37E+06 (1.70E+10) | 2.13E-01 (6.55E+02) | 817.688*** (1319.645) | 1.07E+01*** (4.01E+00) |
| Low middle population | 5.872E+06 (1.540E+10) | 1.88E+06 (2.32E+10) | 2.87E+04 (8.83E+07) | 6322.550*** (7630.428) | 35.092*** (10.404) |
| Middle population | 2.750E+07 (7.200E+10) | 3.27E+08 (4.04E+12) | 1.97E+04 (6.05E+07) | 3964.341*** (4825.223) | 14.953*** (6.472) |
| Big population | 8.650E+07 (1.890E+12) | 1.36E+08 (1.68E+12) | 5.03E-03 (1.55E+01) | 3861.449*** (4814.692) | 12.274*** (5.342) |
| GDP PER CAPITA |  |  |  |  |  |
| Middle income | 0.865 (0.165) | 1.076 (0.171) | 1.114 (0.217) | 1.367 (0.401) | 2.453* (1.082) |
| Upper middle income | 21.068** (22.119) | 1.133 (0.267) | 1.820 (0.488) | 1.561 (0.518) | 1.988 (0.780) |
| High income | 39.235** (43.975) | 0.012** (0.016) | 1.654* (0.766) | 2.062 (0.765) | 3.915*** (1.485) |
| NOCAGE | 1.067 (1718.318) | 1.030 (0.054) | 1.193 (0.848) | 0.917** (0.026) | 1.006 (0.004) |
| ASSOCIATION |  |  |  |  |  |
| Africa | 1.120E-08 (2.060E-04) | 49.169*** (44.602) | 0.000 (0.001) | 1.020 (0.901) | 6.694E-01 (2.070E-01) |
| Asia | 1.737E-01 (2.014E+04) | 22.257 (44.588) | 0.000 (0.001) | 0.000 (0.004) | 4.482E-03*** (3.847E-03) |
| ConSudAtle | Empty | 810.894*** (827.938) | 0.000 (0.000) | 39.063* (62.487) | 0.020*** (0.006) |
| NACAC | 0.000 (0.000) | 253.177*** (310.986) | 0.093 (2.309) | 1.403 (1.214) | 0.839 (0.256) |
| Oceania | omitted | 2.56E+08 (3.17E+12) | empty | empty | 0.418** (0.115) |
| DISCIPLINE GROUP |  |  |  |  |  |
| Sprint | 9.769*** (3.308) | 9.299*** (1.711) | 7.701*** (1.245) | 6.125*** (0.812) | 20.766*** (2.313) |
| Middle distance running | 7.509*** (2.614) | 5.673*** (1.089) | 3.778*** (0.642) | 2.942*** (0.411) | 10.693*** (1.236) |
| Long distance running | 4.112*** (1.417) | 2.219*** (0.411) | 1.629** (0.268) | 1.867*** (0.249) | 3.112*** (0.337) |
| Hurdles and Steeple chase | 4.701*** (1.614) | 3.577*** (0.659) | 2.729*** (0.444) | 2.733*** (0.362) | 7.162*** (0.780) |
| Jumping | 5.881*** (1.982) | 4.189*** (0.753) | 3.614*** (0.572) | 3.561*** (0.459) | 9.488*** (1.010) |
| Throwing | 8.905*** (2.983) | 4.311*** (0.774)) | 3.191*** (0.506) | 4.128*** (0.532)) | 9.261*** (0.985) |
| Cut1 | 27.506 (29142.36) | 26.467 (12379.02) | 10.843 (3073.191) | 8.850 (1.096) | 5.123 (0.607) |
| Cut2 | 30.698 (29142.36) | 34.298 (12379.02) | 16.615 (3073.191) | 13.011 (1.096) | 9.422 (0.613) |
| Number of observations | 12,760 | 12,760 | 13,020 | 12,560 | 12,740 |
| AIC | 4159.182 | 6794.857 | 8558.342 | 12288.04 | 17415.4 |
| Pseudo R² | 0.415 | 0.535 | 0.475 | 0.383 | 0.277 |

**Notes:** Dependent variable is HOSTINGS; Method is ordered logistic regression with country dummies to account for the fixed effects-character of the data. Coefficients for country dummies are not reported. a. Displayed are odds ratios (standard errors in bracket). b. Reference category is ‘Very low WPEI’. c. Reference category is ‘2001’. d. Reference category is ‘Christianity’. e. Reference category is ‘Europe’. f. Reference category is ‘Very small population’. g. Reference category is ‘Very low income’. h. Reference category is ‘Walk’.
*** p<0.001, ** p<0.01, * p<0.05, ^†^p<0.1.
